# PSA-Responsive Aptamer-Based Switchable Aggregates of Ultrasmall Gold Nanoparticles

**DOI:** 10.3390/s26010033

**Published:** 2025-12-20

**Authors:** Giulia Matteoli, Pasquale Mastella, Elisa Ottalagana, Riccardo Nifosì, Luca Bellucci, Fabio Beltram, Giovanni Signore, Stefano Luin

**Affiliations:** 1 NEST Laboratory, Scuola Normale Superiore, Piazza San Silvestro 12, 56127 Pisa, PI, Italy; pasquale.mastella@sns.it (P.M.); elisa.ottalagana@sns.it (E.O.); riccardo.nifosi@nano.cnr.it (R.N.); luca.bellucci@nano.cnr.it (L.B.); fabio.beltram@sns.it (F.B.); 2 Fondazione Pisana per la Scienza ONLUS, Via Ferruccio Giovannini 13, 56017 San Giuliano Terme, PI, Italy; giovanni.signore@unipi.it; 3 Present Address: Institute of Clinical Physiology-Italian National Research Council, Via Giuseppe Moruzzi 1, 56124 Pisa, PI, Italy; 4 NEST, Istituto Nanoscienze-CNR, Piazza S. Silvestro 12, 56127 Pisa, PI, Italy; 5 Biochemistry Unit, Department of Biology, University of Pisa, via san Zeno 51, 56123 Pisa, PI, Italy

**Keywords:** prostate cancer, nanoparticles, aptamers, cancer diagnostics, responsive nanosystems, dynamic light scattering (DLS)

## Abstract

Prostate-specific antigen (PSA) is a key biomarker for the early detection of prostate cancer recurrence following surgical treatment. In this study, we present a PSA-responsive, aptamer-based switchable aggregate system, named AS2-US-AuNP-Aggregate, composed of ultrasmall gold nanoparticles (US-AuNPs) linked by (partially) pairing oligomers that selectively disassemble in the presence of PSA. The system was optimised also using a previously developed in silico routine and is designed for enhanced detection capabilities and for supporting in vivo applicability. We measured the sizes of the nanosystems by dynamic light scattering (DLS) and their extinction spectra, also in the presence of PSA in simple buffers, in the presence of DNaseI, and under blood-mimicking conditions (filtered plasma), obtaining a response down to 10 fM PSA in buffers and to 1 pM in filtered plasma. Our findings highlight the potential of aptamer-based nanoparticle aggregates as a basis for user-friendly diagnostic tools. Additionally, we discuss key optimisation strategies to further advance their development for in vivo diagnostic applications.

## 1. Introduction

Prostate cancer (PCa) is the second most diagnosed cancer in men, with approximately 1.4 million new cases per year [1]. One possible treatment for PCa is radical prostatectomy (RP), a surgical procedure involving the complete removal of prostate tissue. Following RP, the prostate-specific antigen (PSA) serum level is monitored over time to detect disease relapse. PSA is a secreted protein with a molecular weight of 33 kDa, expressed in both normal and cancerous prostatic tissue, and should not be present in blood after total prostate resection. However, the presence of residual tumour margins after surgery can lead to a cancer relapse with a progressive increase in serum PSA level. A biochemical recurrence (BCR) occurs when PSA levels rise above 0.2 ng/mL (6 pM, the current critical threshold) in two consecutive measurements, and this is highly indicative of tumour relapse. Using this threshold (defined also as nadir point [2]), approximately 23% of patients experience BCR within five years after surgery.

The development of novel ultrasensitive PSA assays (USPSAs), defined as assays with a limit of detection (LoD) below 1 pM [3], has significantly improved the ability to detect early rises in PSA levels. This advancement enables earlier BCR detection following RP, allowing for improved patient management. Furthermore, the introduction of USPSAs has led to the proposal of alternative nadir points, such as 300 fM (0.01 ng/mL) [4] and 150 fM (5 pg/mL) [5]. However, USPSAs available in clinical settings still have limitations, including the need for specialised laboratories, high costs, and lack of standardisation across different platforms, leading to variability in the results. In parallel, the development of commercial point of care (PoC) PSA tests based on gold nanoparticles, such as Lateral Flow Assay (LFA)-based ones, has provided portable, easy-to-use, non-invasive assays that deliver rapid results. However, these tests suffer from a higher LoD range of 9 pM—3 nM [6].

In this context, there is a growing interest in the development of more sensitive assays. An innovative nanoparticle-based strategy for diagnostic and prognostic applications is based on an in vivo use of triggerable nanostructured systems with an ex vivo readout. These systems are injected into living organisms, and upon recognition of a biomarker, they disassemble, releasing renal-clearable reporters, which can be rapidly quantified in urine [7,8,9,10,11,12,13,14,15,16]. These systems typically exploit enzymatic biomarkers such as metalloproteinases or β-glucuronidases, which are overexpressed in tumours. Although aptamer-based gold nanoparticles have been studied for imaging purposes [17], only one study applied the discussed rationale for the in vivo detection of a protein biomarker exploiting aptamers, using quantum dots (QDs) as the renal-clearable reporters for a direct colorimetric read out in urine [12]. This approach has proven highly effective in enabling earlier diagnoses with respect to traditional in vitro assays, due to increased tumour environment accessibility and longer retention. Various platforms have been explored for this purpose, including polymeric nanoparticles (NPs), fluorescent probes, and inorganic NPs. Among them, inorganic-NP-based systems, particularly the ones using metal nanoparticles (MNPs), offers notable advantages. These systems can be designed as larger nanostructures (typically 100–200 nm) to exploit the enhanced permeability and retention (EPR) effect for accumulation in diseased tissues, which can be increased if they comprise some targeting element. Subsequently, they can release ultrasmall nanoparticles with a hydrodynamic diameter (HD) < 6 nm, which are efficiently cleared through the kidneys via rapid renal excretion.

In this study, we present an activatable aptamer-nanoparticle-based structure responsive to PSA, named AS2-US-AuNP-Aggregate, composed of aggregated ultrasmall gold nanoparticles (US-AuNPs) having a suitable size for renal clearance. This system is based on a switchable nucleotide architecture composed by two (partially) complementary sequences attached to different US-AuNPs; upon hybridisation, these architectures act as linkers amongst the US-AuNPs. One of the oligonucleotides incorporates an aptamer specific for PSA, according to the same design principles of a similar nanostructure previously developed by our group using bigger AuNPs [18,19]. Upon biomarker recognition, the system releases, or is disassembled into, single US-AuNPs. The nanostructure development was initiated by designing switchable sequences through an in silico approach. We then tested the disassembly of the produced AS2-US-AuNP-Aggregates in the presence of different concentrations of PSA; the primary tool used to monitor this disaggregation in vitro was dynamic light scattering (DLS), which allows for determining the decrease in the average size of the nanostructures in the solution. Moreover, since this work represents a preliminary study to assess the feasibility of in vivo applications of this system, we evaluated the stability and the responsiveness of the AS2-US-AuNP-Aggregate nanostructures in the presence of blood components. This step is crucial, because physiological fluids, such as plasma, contain biomolecules that may compromise the properties of the nanostructures. By investigating the behaviour of the system in these complex conditions, we aimed to evaluate its functionality in biologically relevant environments, thus providing key insights into its suitability for translational applications in real-time, non-invasive diagnostics.

## 2. Materials and Methods

### 2.1. Materials and Instrumentation

Nucleotidic and aptameric sequences were purchased from Metabion (Planegg, Germany). The native human prostate-specific antigen (PSA) was obtained from Abcam (Cambridge, UK). Fluorophores used for ssDNA functionalisation were NHS-Rhodamine (5/6-carboxy-tetramethyl-rhodamine succinimidyl ester) (Thermo Scientific™,) and Atto580Q-NHS-ester (ATTO-TEC GmbH, Siegen, Germany). DNaseI Solution (Thermo Scientific™) used for nuclease stability experiments was purchased through Thermo Fisher Scientific (Milan, Italy). Human Plasma was obtained from Biowest (Nuaillé, France) and Human Female Plasma from Aurogene Srl (Rome, Italy). The chemicals used in this study were the following: Gold(III) chloride (HAuCl_4_) trihydrate, Sodium Borohydride, Tris(2-carboxyethyl)phosphine hydrochloride (TCEP), 3,3′,5,5′-Tetramethylbenzidine (TMB), Horseradish Peroxidase-streptavidin (HRP-streptavidin), Phosphate-Buffered Saline (PBS) and Bovine Serum Albumin (BSA), all from Sigma-Aldrich^®^ (Merck KGaA, Darmstadt, Germania). For the Enzyme-Linked Oligonucleotide Assay (ELONA), we used Corstar^®^ 96 well assay flat bottom plates (Corning, Milan, Italy), and colorimetric readings were performed with the Infinite^®^ PRO 200 (TECAN, Männedorf, Switzerland) instrument. Fluorescence recovery analysis was performed with a Cary Eclipse Fluorescence Spectrometer (Agilent, Milan, Italy). DLS, performed with a Zetasizer Nano ZS (Malvern Panalytical, Lissone, MB, Italy), and UV-Vis spectra, performed with a Cary 3500 UV-Vis spectrophotometer (Agilent, Milan, Italy), were used for nanostructure characterisation. DLS was set with water as the solvent and gold as the material of the nanoparticles. DLS for Zeta potential measurements was set in automatic mode. All measurements were performed with a backscatter measurement angle θ = 137°.

### 2.2. Enzyme-Linked Oligonucleotide Assay (ELONA)

The aPSA [20], AS2 [21], and As2-tail sequences (see Supplementary Table S1) were tested with ELONA. A 96-well assay plate was incubated with 250 ng of PSA (diluted in 100 μL of 0.1 M NaHCO_3_ pH 9.6 buffer) at 37 °C for one hour with agitation. Then, the PSA solution was removed, and without additional washing, the well was incubated for 2 h at room temperature with 200 μL of Blocking Solution (BSA 3% + 100 nM of an equimolar mixture of the coating ssDNA sequences hlyQF, hlyQR, L23SQF, and L23SQR) [22]. After removal of the Blocking Solution, 100 μL of solutions containing the biotinylated-sequences were added without additional washing. The folded biotinylated-sequences (aPSA-biotin, AS2tail-biotin, and annealed AS2tail-biotin:RevAS2) were prepared in 1:2 serial dilutions in PBS buffer supplemented with 5 mM MgCl_2_, ranging from 500 nM to 3.9 nM plus a blank control (without DNA). Each condition was prepared in triplicate, for a total of 24 wells per sequence, and the plate was incubated for 1 h at 37 °C with gentle shaking. The solution was then removed, and the wells were washed once with 200 μL of Washing Solution (PBS Tween-20 0.05% (*v*/*v*)). Next, 100 μL of the enzyme conjugation solution (HRP-streptavidin diluted 1:20,000 in PBS BSA 1%) was added and incubated for 1 h with gentle shaking at room temperature. The enzyme-conjugation solution was removed and each well was washed six times with 200 μL of Washing Solution. Then, 100 μL of TMB solution was added, and the plate was incubated from 3 to 20 min, until the solution developed a visible blue colour, at which point 100 μL of TMB stop solution (0.16 M sulphuric acid) was added. A multichannel pipette was used to dispense and remove the solutions with minimal time delay. Absorbance values were read at 450 nm using the Infinite^®^ PRO 200 plate reader (TECAN, Männedorf, Switzerland), and the response curve was generated after applying blank subtraction (samples without aptamer).

### 2.3. Switchable Nucleotide-Sequence Development

The in silico protocol applied to investigate the interaction of the AS2 aptamer and PSA was developed and described in a previous work [19]. This protocol was used to predict which nucleotides of the aptamer contact PSA and to estimate their respective energy contributions to the binding interaction. Briefly, after obtaining the 3D structure of the aptamer, we performed a flexible docking interaction and post-docking analysis between the aptamer and the target protein. The crystallographic structure of the PSA protein used for the simulation corresponds to the Protein Data Bank (PDB) code 2ZCK.

The NUPACK online tool (NUPACK3 version [23]) was used to predict the secondary folding and thermal stability of coupled sequences (duplexes) [23,24]. Thermal stability was calculated between 30 and 70 °C using a saline concentration of 150 mM of NaCl and 25 mM of Mg^++^. In silico melting temperatures (T_m_) of the complexes were estimated using a concentration for each oligonucleotide of 100 nM: T_m_ was considered the temperature at which the concentration of the duplexes lowered to 50 nM. Experimental melting temperatures of produced coupled oligonucleotides (AS2-tail:RevAS2 and AS2tail-ctrl:RevAS2-ctrl; see Supplementary Table S1) were measured by performing absorbance reading at 260 nm. Firstly, sequences were annealed in Annealing Buffer (50 mM Tris-HCl, pH 7.5, 150 mM NaCl, 25 mM MgCl_2_) at a concentration of 100 nM for both coupling oligonucleotides with the following thermal steps: 2 min at 95 °C and then cooling at 12 °C with a temperature ramp of −2 °C/min. For melting curve recording, absorbance values of the annealed DNA solutions were recorded at 260 nm from 25 to 75 °C, with temperature steps of 1 °C and with a temperature rate of +2 °C/min. For in vitro analysis, the melting temperature of the annealed sequences was determined as the one at the maximum value of the first derivative of the absorbance (A) with respect to the temperature (T) of the recorded melting curve (dA/dT).

### 2.4. US-AuNP Synthesis and Functionalisation

US-AuNPs were synthetised following a protocol for obtaining 2 nm diameter nanoparticles [25] and characterised for size (DLS) and plasmonic properties (absorbance reading). Briefly, 375 μL of a 4% HAuCl_4_ solution and 500 μL of 0.2 M K_2_CO_3_ were added to 100 mL of cold Millipore water, while stirring. Then, five 1 mL aliquots of 0.5 mg/mL sodium borohydride (NaBH_4_) solution were added to the reaction solution while rapidly stirring. US-AuNP concentration was calculated using a molar extinction coefficient of 6.5 × 10^5^ cm^−1^ M^−1^ at a wavelength of 510 nm [26]. US-AuNPs were decorated with thiolated sequences (i.e., sequences modified by C3-SS or C6-SS thiol modifier) reduced by incubation of 30 μL of oligonucleotide solution (100 μM) with 3 μL of 100 mM TCEP and 2 μL of Acetate Buffer 100 mM pH 5.2 for 1 h at room temperature. Reduced sequences were purified with Amicon spin 3 KDa filters and quantified by absorbance reading at 260 nm. For US-AuNP functionalisation, 1 nanomole of reduced ssDNA was incubated with 100 μL of the US-AuNP solution with shaking overnight at room temperature. The day after, 500 mM Tris-acetate (pH 8.2) buffer and 1M NaCl were gently added dropwise to each vial to obtain a final Tris-acetate concentration of 5 mM and NaCl final concentration of 300 mM. The reaction vials were stored with shaking for one additional night. ssDNA-decorated US-AuNPs (ssDNA-US-AuNPs) were purified with Amicon 100 KDa Filters and suspended in 300 mM NaCl, 25 mM Tris acetate, pH 8.

The ssDNA/particle ratio was estimated with UV-Vis measurements: the concentration of the US-AuNPs was obtained by absorbance at 510 nm considering an extinction coefficient of 6.5 × 10^5^ cm^−1^ M^−1^ [26], and the concentration of the ssDNA was determined considering the absorbance of the ssDNA-US-AuNPs at 260 nm after subtracting the contribution of the US-AuNPs at such wavelength; this was calculated knowing the US-AuNP concentration, from the known extinction coefficient of the bare US-AuNPs at 260 nm, which can be determined by the ratio of the absorbance of a US-AuNP sample at 260 nm and its concentration, determined as described above. The obtained value was used to determine the oligonucleotides concentration of the sample by knowing the extinction coefficient of the oligonucleotides at 260 nm as specified by the manufacturer. Finally, the ssDNA/particle ratio was calculated by dividing the obtained ssDNA concentration by the US-AuNP concentration.

### 2.5. US-AuNP-Aggregate Assembly and Characterisation

To prepare the AS2-US-AuNP-Aggregate, equal volumes of two separately synthesised batches—RevAS2-US-AuNPs and AS2-tail-US-AuNPs—were mixed at a 1:1 ratio, stored at 4 °C overnight while stirring, and purified by centrifuging the solution at 1000× *g* for 2 min; the nanoparticle pellet was resuspended in 150 mM NaCl 5 mM Tris-acetate pH 8.2 buffer solution. The AS2ctrl-US-AuNP-Aggregate was prepared using the same protocol, mixing AS2-tail-ctrl-US-AuNPs with RevAS2-ctrl-US-AuNPs. DLS and UV-Vis spectroscopy were employed to characterise the size distribution and optical properties of both aggregates. The plasmonic peaks of the aggregates are determined by identifying the wavelengths at which the first derivative of the absorbance with respect to wavelength equals zero, after applying a five-point average smoothing. Thermal stability was assessed by monitoring the changes in hydrodynamic diameter at DLS across a temperature range of 30 °C to 75 °C.

### 2.6. AS2-US-AuNP-Aggregate PSA Response Characterisation in PBS

Stability and kinetic analysis of the AS2-US-AuNP-Aggregate in the presence or absence of different concentrations of PSA was conducted for about 30 min at 37 °C using DLS (set to perform 15 measurements in automatic mode). For these studies, the aggregate was used at a final concentration corresponding to an absorbance (with 1 cm light path) of 0.02–0.03 at the plasmon peak. Control experiments were performed with the AS2ctrl-US-AuNP-Aggregate under identical conditions. PSA was tested initially at 1 pM and 1 nM. DLS data were analysed using the instrument software with the following settings: material set as gold (refractive index [RI] = 0.20; absorption = 3.320) and dispersant set as water (RI = 1.330; viscosity = 0.6864 mPa⋅s). Data processing was carried out under the “general purpose” analysis mode, and the resulting size distributions were reported as provided by the software. Additionally, we further analysed the DLS-provided correlograms as explained in the following. The correlogram characterises the fluctuations of the intensity I(t); in particular, its autocorrelation function $G^{2}\left(\tau \right)$ is defined as $G^{2}\left(\tau \right)=\langle I\left(t\right)I(t+\tau )\rangle$, where the angled bracket indicates an average over time t. $G^{2}\left(\tau \right)$ can usually be written as

(1) $$G^{2}\left(\tau \right)=A\left(1+B{\left|g^{1}\left(\tau \right)\right|}^{2}\right),$$

where $g^{1}\left(\tau \right)$ is the normalised electric field autocorrelation function, and *A* and *B* are parameters which could depend on instrument and sample. If the colloid is not monodispersed, $g^{1}\left(\tau \right)$ becomes a sum of several exponential decays or an integral representing a continuous distribution of hydrodynamic diameters. The DLS software allows for exporting a correlation coefficient derived from the $G^{2}\left(\tau \right)$ by subtracting the long lag-time background $\langle I{\left(t\right)\rangle }^{2}$, assuming the intensity remains relatively constant during the measurement, and by applying a normalisation. This correlation coefficient is proportional to ${\left|g^{1}\left(\tau \right)\right|}^{2}$. We fit this function with a squared multiexponential function

(2) $${\left|g^{1}\left(\tau \right)\right|}^{2}={\left(\sum_{i=1}^{N}c_{i}e^{-\frac{\tau }{\tau_{i}}}\right)}^{2};$$

the number of components N was set to 2 or 3, with constraints sometimes imposed on possible ranges of $\tau_{i}$. Each $\tau_{i}$ corresponds to a hydrodynamic diameter given by

(3) $$d_{i}=\frac{16\pi kTn^{2}{\left(\sin\left(\theta /2\right)\right)}^{2}\tau_{i}}{3\eta \lambda^{2}}\approx 0.464\;\tau_{i}\left(µs\right)\;\mathrm{nm}.$$

In our setup, T is the temperature, k the Boltzmann constant, n the refraction index, the viscosity of the medium, and θ = 137° the already specified measurement angle. For the experiment on AS2-US-AuNP-Aggregates incubated with PSA, the fitting constraints were τ_1_, 0–250 µs (corresponding to d_1_ ≤ 116 nm); τ_2_, 250–700 µs (d_2_ in the 116–325 nm range); and τ_3_ > 1 ms (d_3_ > 464 nm). The AS2-US-AuNP-Aggregate was then tested by incubating it with PSA in a concentration range between 1 fM and 100 nM. The AS2-US-AuNP-Aggregate was also incubated with BSA at a concentration range from 1 fM to 500 μM. The AS2ctrl-US-AuNP-Aggregate was incubated in the same conditions. These tests were performed at a temperature of 37 °C. If not otherwise stated, measurements were taken 15 min after the addition of PSA to the aggregate suspension and recorded for about 5 min. The number-weighted average size of the aggregates was monitored by DLS measurements; we also measured the absorbance spectra.

### 2.7. Stability of the AS2-US-AuNP-Aggregate to DNaseI Activity

An AS2-US-AuNP-Aggregate solution at a DNA final concentration of 200 nM was incubated with DNaseI at a concentration of 0.3 U/mL. DNA digestion was monitored for 14 h by measuring AS2-US-AuNP-Aggregate size and count rate with DLS every 60 min starting immediately after the addition of DNaseI.

### 2.8. Effect of Plasma Nucleases on Annealed AS2-Tail-Rhodamine:RevAS2-atto580Q Sequences Analysed by Förster Resonant Energy Transfer (FRET)

The aminated oligonucleotides AS2-tail-NH_2_ and RevAS2-NH_2_ (see Supplementary Table S1) were labelled, respectively, with NHS-Rhodamine and atto580Q-NHS-ester. Labelling reaction was performed overnight at room temperature in PBS 10× using a 10-fold molar excess of the fluorophore. The labelled ssDNAs were isolated by fraction collection in High-Performance Liquid Chromatography (HPLC) using a DNAPac™ PA100 (Thermo Scientific™) column, and Tris HCl 20 mM pH 7.6 (eluent A) and Tris HCl 20 mM NaCl 1 M pH 7.6 (eluent B) as mobile phases with a gradient from 30% to 100% of B mobile phase in 20-min run. Collected fractions were concentrated in MilliQ water using Amicon 3 KDa filters, previously coated with BSA 1%. AS2-Rhodamine and RevAS2-atto580Q were annealed at equal molar ratios as described earlier. A solution of AS2-Rhodamine:RevAS2-atto580Q 20 nM was incubated with human plasma (Biowest, France) diluted 1:8 at a temperature of 37 °C. Fluorescence measurements were taken at 15-min intervals for 10–30 h. The fluorometer was set with an excitation wavelength of 540 nm with a slit aperture of 10 nm and emission wavelengths between 550 and 750 nm with a slit aperture of 5 nm. The photomultiplier tube (PMT) voltage was maintained at 900 V throughout the measurements. Rhodamine fluorescence intensity was calculated as the area under the curve (AUC) of the emission spectrum after background subtraction (refer to Supplementary Result S3) in the wavelength range 570 to 610 nm. For the stability of AS2-Rhodamine:RevAS2-Atto580Q towards DNaseI, a solution containing 200 nM of AS2-Rhodamine:RevAS2-Atto580Q was incubated with DNaseI 0.3 U/mL. Fluorescence measurements were conducted at 60-min intervals over a 14-h period and quantified as before. Averages and integrals have been calculated using Microsoft Excel; fit of the backgrounds has been performed using OriginPro 9 (version 90E; OriginLab Corporation, Northampton, MA, USA), and calculation of additive and multiplicative constants for the background (see Supplementary Result S3), background subtraction, and integration of the spectra have been automatised using a home-made script in MATLAB R2017b (version 9.3.0.713579; The Mathworks, Inc., Natick, MA, USA).

### 2.9. US-AuNP-Aggregate Interaction with Filtered Human Plasma

Human female plasma (pooled; TCS Biosciences, UK) was processed as follows: plasma was first centrifuged at 3000× *g* for 30 min at 4 °C to remove cells and cellular debris. The resulting supernatant was centrifuged at 10,000× *g* for 1 h at 4 °C, and the supernatant was filtered using SterilFlip^®^ 0.22 μm filter to remove microvesicles. Then, the obtained liquid was centrifuged at 33,000× *g* for 1 h and 30 min at 4 °C to eliminate exosomes and larger plasma complexes. The obtained supernatant was finally filtered using an Amicon 100 kDa filter at 12,000× *g* for 15 min to remove large proteic and lipidic complexes. Plasma was characterised using DLS at every purification step. The stability and the response to PSA presence of the AS2-US-AuNP-Aggregates in the presence of plasma was evaluated by incubating them at a concentration causing an optical density of 0.1 at the plasmonic peak with the filtered plasma diluted 1:6 at 37 °C. The aggregate stability was assessed over a 2-h duration through DLS. The response to PSA was tested at PSA concentrations of 10 fM, 100 fM, 1 pM, and 10 pM. The reaction kinetics were monitored using DLS over a 20-min interval.

### 2.10. Statistical Tests

All data are reported as mean ± standard deviation (in tables) or standard error (in figures). All the data sets were analysed with *t*-test comparisons. Significance was attributed to a *p* value less than 0.05. Specifically, in figures, significance is represented as follows: *p* < 0.05 is *, *p* < 0.01 is **, and *p* < 0.001 is ***. A linear regression analysis y=mx+q was performed for the behaviour of the averaged sizes and of the plasmon peak positions as a function of the log_10_ of PSA concentrations expressed in fM. From the fit residuals, an average standard deviation $\sigma_{y}$ for the analysed quantity y (size or plasmon peak position) was estimated as follows:

(4) $$\sigma_{y}=\sqrt{\frac{\sum_{i=1}^{N}{\left(y_{i}-{\bar{y}}_{i}\right)}^{2}}{dof}},$$

where $y_{i}$ is the experimental value of y at the *i*-th value of PSA concentration, ${\bar{y}}_{i}$ is the corresponding value obtained by the fit, N is the number of points in the fit, and dof=N−2 is the number of degrees of freedom for the linear fit. A sort of limit of detection (LoD) parameter was estimated as follows:

(5) $$\mathrm{LoD}=10^{3.3\frac{\sigma_{y}}{m}}.$$

## 3. Results

### 3.1. PSA-Binding Aptameric Sequence Design and Validation

**Figure 1:**
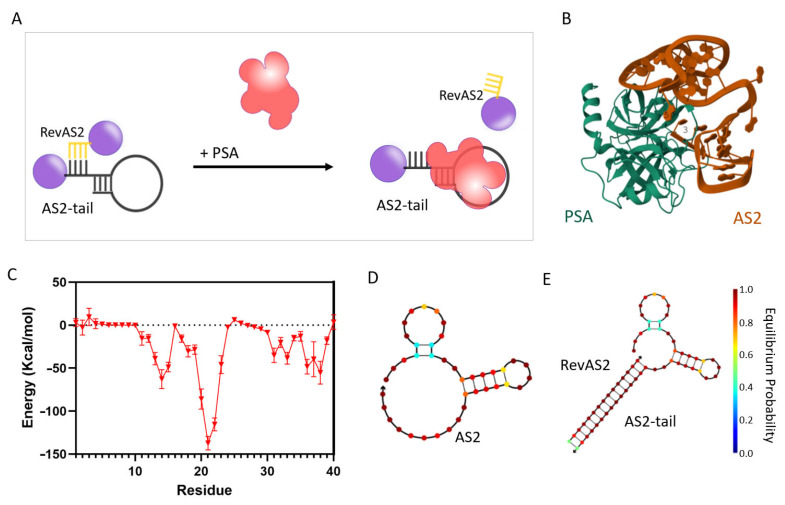
(**A**) Schematic of the PSA sensing reaction of the AS2-US-AuNP-Aggregate. The AS2-US-AuNP-Aggregate is composed of US-AuNPs (purple spheres; for simplicity, only two are shown here, but since there are more ssDNAs per US-AuNP, there are usually aggregates composed of many US-AuNPs) and decorated either with the partial complementary sequences RevAS2 or with the AS2-tail ssDNAs. The PSA interaction with the AS2 aptamer within the AS2-US-AuNP-Aggregate triggers the release of RevAS2-US-AuNPs. (**B**) Docking model of the complex between the AS2 aptamer ssDNA (orange) and the PSA human protein (aqua green), obtained by molecular dynamic simulations exploiting various software, including the HADDOCK webserver [19], and visualised using Visual Molecular Dynamics (VMD). (**C**) Potential energy contribution of each nucleotide in the AS2 sequence to the binding energy of the aptamer AS2 with the PSA protein (red triangles and line). The standard deviation reflects the variability of these energy contributions over the course of a 4 ns simulation. (**D**,**E**) Most probable secondary folding of the AS2 aptamer (**D**) and of the AS2-tail:RevAS2 duplex (**E**) obtained with the NUPACK online tool. The colour code for the probability of base pairing at equilibrium (i.e., the probability of the corresponding base pair forming) according to the colour scale bar reported on the right. The simulation was performed with NUPACK online tool at the following conditions: DNA concentration 100 nM, temperature 37 °C, NaCl concentration 0.15 M, and Mg^2+^ concentration 0.025 M.

For the development of the US-AuNP-Aggregate, we evaluated two previously published aptameric sequences, aPSA [20] and AS2 [21]. The binding ability and affinity of these aptamers was assessed by direct ELONA [27] (Section S1, Figure S1). In our experiments, the aPSA aptamer did not exhibit any detectable binding response (Figure S1A). In contrast, the AS2 sequence demonstrated significant binding starting at a concentration of 250 nM (*t*-test, *p* < 0.05), confirming its binding ability under our experimental conditions, which included a temperature of 37 °C and physiological saline concentration (Figure S1B, black line; Section S1). Considering these results, we selected the AS2 aptamer for the development of the switchable nucleotidic architecture. This construct relies on two DNA sequences: AS2-tail, which includes the AS2 aptamer flanked by a tail of additional bases, and RevAS2, which is complementary to a portion of AS2-tail (as better explained below) and is designed to be released upon PSA recognition (Figure 1A, Table S1).

In order to achieve this, the design of RevAS2 is critical: in particular, the selection of the AS2 bases annealing to it is essential for enabling the switching mechanism. These specific AS2 nucleotides hybridising with RevAS2 (referred to as “trigger nucleotides”) should interact with PSA with an energy high enough to support the triggering of the system activation but should not be part of the highest-affinity binding domains. In this way, these high-affinity regions can start the recognition of PSA, bringing also the trigger nucleotides into close proximity to the domain of the protein where they bind. This spatial arrangement should facilitate the complete aptamer engagement with PSA, thereby inducing de-hybridisation of the duplex structure. However, experimentally identifying the most effective trigger nucleotides can be labour-intensive. To streamline this, we analysed the molecular interactions between the AS2 aptamer and PSA using a previously developed molecular docking approach [19]. As shown in Figure 1B, the energy contributions of individual nucleotides calculated from the docking results (Figure 1C) revealed a key interaction domain spanning bases 17 to 24, part of a stem-loop structure spanning bases 17–30 (see the secondary structure prediction in Figure 1D). Additional interactions derive from nucleotides 11 to 15, which form another part of a prominent stem-loop structure spanning bases 4–14 at the 5′ terminus (Figure 1D), and from the 3′ end, between bases 31 to 39, in a region that is unstructured in the free aptamer. Based on these findings, RevAS2 was designed to hybridise this last region, in particular with nucleotides 32–40 at the 3′ terminus of the AS2 aptamer. Comparative secondary structure predictions at 37 °C for the unmodified AS2 aptamer and the AS2-tail:RevAS2 duplex (Figure 1D,E) suggested the maintenance of the aptamer native folding, which is essential for PSA binding. A control duplex (AS2ctrl:RevAS2ctrl) was designed using a scrambled sequence for the aptamer (Figure S2A,B). The full list of sequences used in this study is provided in Table S1.

**Figure 2:**
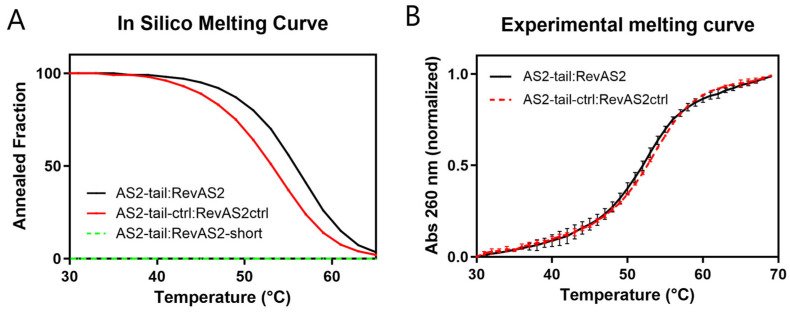
Melting curves of AS2-tail:RevAS2 (in black) and AS2-tail-crtl:RevAS2-ctrl (in red) annealed sequences determined in silico with NUPACK (**A**) and in vitro by absorbance measurements (**B**). Panel (**A**) also includes results for AS2-tail mixed with RevAS2-short, i.e., the RevAS2 lacking the nucleotides that should interact with PSA (green dashed line). In (**B**), absorbance is normalised between 0 and 1 at the lowest and highest measured temperatures.

Since the sensing reaction is designed to work at body temperature (37 °C), we assessed the thermal stability of the AS2-tail:RevAS2 and AS2-tail-ctrl:RevAS2-ctrl duplexes. Melting temperatures (T_m_) were calculated during the duplex design stage using NUPACK simulations, which predicted a T_m_ of 56 °C for the AS2-tail:RevAS2 duplex and 53 °C for the AS2-tail-ctrl:RevAS2-ctrl duplex (Figure 2A, Table S2). To estimate the stability of AS2-tail:RevAS2 upon PSA binding, we predicted the melting curve for a RevAS2-short strand, obtained from RevAS2 removing the nucleotides complementary to the aptamer, hybridised with the complete AS2-tail. This resulted in no duplex formation within the considered temperature range, suggesting that the duplex would not remain stable upon PSA binding (Figure 2A, green dashed line). The experimentally measured T_m_ was 52 °C for the AS2-tail:RevAS2 duplex and 53 °C for AS2ctrl:RevAS2ctrl (Figure 2B, Table S2), consistent with the simulated values.

The affinity of the AS2-tail:RevAS2 duplex for PSA was confirmed by ELONA, obtaining a significant signal starting from a dsDNA concentration of 250 nM (*t*-test, *p* < 0.05). These findings demonstrate that AS2 retains its binding capability when incorporated into the duplex structure, maintaining comparable affinity to the native aptamer sequence under physiological conditions (Figure S1B, Supplementary Result S1).

### 3.2. Ultrasmall-AuNP Functionalisation and Characterisation

**Figure 3:**
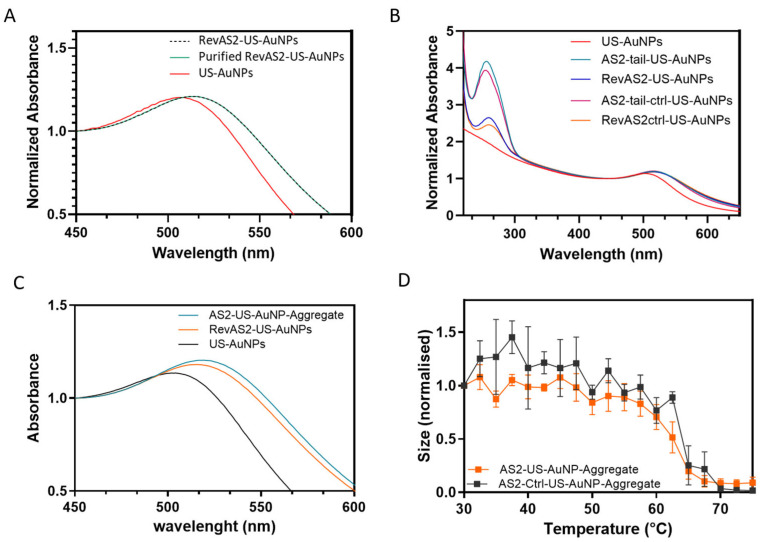
(**A**) Exemplary extinction spectra normalised at 1 at λ = 450 nm of US-AuNPs (red line) and of RevAS2-US-AuNPs before and after the last purification step (black dashed and green solid lines, respectively). (**B**) Full UV-Vis extinction spectra, normalised at 1 at λ = 450 nm, of non-functionalised (red line) and ssDNA-functionalised US-AuNPs (orange, pink, blue, and dark cyan line, as better specified in the legend). (**C**) Representative extinction spectra normalised at λ = 450 nm, showing the plasmonic peak, of single non-functionalised AuNPs (black line), ssDNA-AuNPs (orange line), and AS2-US-AuNP-Aggregates (dark cyan line); batches of AuNPs and RevAS2-US-AuNPs are different than in panel A. (**D**) Melting curves of the AS2-AuNP-Aggregate (orange squares and line) and of the control AS2-CTRL-AuNP-Aggregate (black squares and line) as obtained from the DLS-measured number-weighted average size of the Aggregates, normalised at 1 at the lowest measured temperature. The measurement is reported as mean values of a triplicate (n = 3), and the error bars are standard errors.

US-AuNPs, synthetised as explained in the experimental section, were characterised for both size and zeta-potential by DLS (Table S3) and for plasmonic properties by absorbance reading (Figure 3A–C and Table S3). The obtained US-AuNPs showed an hydrodynamic diameter with a number-weighted average size of 4.6 ± 0.2 nm; a plasmonic peak at 508.0 ± 1.0 nm, consistent with the expected nanoparticle size [26] (Figure 3A, red line); and a zeta-potential of −11.5 ± 1.3 mV (measurements are in triplicate and derived from a single batch preparation of nanoparticles; uncertainty is the standard deviation). The plasmon peak position of the naked US-AuNPs was evaluated 24 h post-synthesis, revealing a slight redshift, an indication of an increased size, thus of a colloidal instability (Figure S3). This increase in size is supported by an increase in the measured intensity-weighted average of the diameters, from 7.4 ± 0.3 nm to 9.3 ± 1.0 nm (although the number-weighted average diameter remained consistent, from 4.5 ± 0.6 to 4.5 ± 0.2 nm). Even if the effect was small, these results suggest that naked US-AuNPs tend to aggregate into larger NPs and highlight the necessity for immediate functionalisation after preparation. Accordingly, in the following syntheses, we immediately functionalised the US-AuNPs with thiolated oligonucleotides (AS2-tail-thio, RevAS2-thio, AS2-tail-ctrl-thio, and RevAS2-ctrl-thio; see Table S1). AS2-tail-, RevAS2-, AS2-tail-ctrl-, and RevAS2-ctrl-US-AuNPs refer to gold nanoparticles functionalised with the respective DNA sequences. ssDNA-US-AuNPs were characterised for size and plasmonic peak before and after the last purification step, revealing no changes in their properties (Figure 3A), and this also supports the absence of aggregation. The obtained ssDNA-US-AuNPs showed an increased hydrodynamic radius (11.9 ± 0.4 nm for AS2-tail-US-AuNPs and 7.7 ± 0.4 for RevAS2-US-AuNPs), a plasmonic peak shifted toward longer wavelengths (517.5 ± 0.3 nm for As2-tail-US-AuNPs and 515.3 ± 0.3 for RevAS2-US-Au-NPs), and a lower zeta-potential (−19.7 ± 0.5 mV for AS2tail-US-AuNPs and −18.8 ± 1.8 mV for RevAS2-US-AuNPs) compared to US-AuNPs. AS2ctrl-US-AuNPs and RevAS2ctrl-US-AuNPs exhibited the same trend (full characterisation data are reported in Table S3). The number of DNA molecules per nanoparticle was calculated by comparing the absorbance spectra of the US-AuNPs with those of the ssDNA-functionalised US-AuNPs (Figure 3B), as explained in the experimental section, obtaining an averaged estimated ssDNA/particle functionalisation ratio of 8.1 ± 2.9.

### 3.3. US-AuNP-Aggregate Assembly and Characterisation

The aggregates were assembled as described in the experimental section. The resulting number-averaged sizes of the aggregates were 189 ± 35.3 nm for the AS2-US-AuNP-Aggregate and 157.5 ± 31.6 nm for the AS2ctrl-US-AuNP-Aggregate. The plasmonic peak fell at 526.5 ± 0.5 nm and 528.3 ± 0.5 nm, respectively (Table S3, Figure 3C). Even if the plasmonic shift was lower than the one expected for full gold NPs with the same diameter as the one measured by DLS, it was positive, as expected for the increase in US-AuNP-Aggregate size. In these aggregates, nanoparticles are linked by an oligonucleotide sequence of about 40 base pairs, resulting in an average interparticle distance of about 12 nm. As previously reported in similar structures [19], this spacing leads to only partial plasmonic electromagnetic coupling between the US-AuNPs and therefore to a reduced plasmonic shift.

Thermal stability studies of the US-AuNP-Aggregates demonstrated that the nanostructures remain stable at physiological temperature and undergo disassembly into individual US-AuNPs upon reaching temperatures a little higher than the T_m_ of the involved duplexes (Figure 3D and Table S2). These results are also in agreement with the fact that aggregate formation is driven by annealing-induced assembly of the DNA-linked nanoparticle. The slightly higher melting temperature of the US-AuNP-Aggregates than the one of the dsDNA alone may be attributed to various factors that introduce complexity and influence the T_m_ when transitioning to DNA-linked nanoarchitectures, such as the DNA surface coverage, its high density on the surface, and the expected multiple dsDNA-linkages between NPs [28].

### 3.4. AS2-US-AuNP-Aggregate Response to PSA

The kinetic behaviour of the AS2-US-AuNP-Aggregates alone and upon PSA introduction are key factors in assessing their suitability for potential practical in vivo applications. In the absence of PSA, the AS2-US-AuNP-Aggregate and the AS2-Ctrl-US-AuNP-Aggregate showed no significant size variation over time, as observed in the DLS measurements, indicating stability over at least 30 min at 37 °C (Figures S4 and S5B). Moreover, although no specific long-time stability analysis was performed, we observed that the aggregates remained functional for at least one week if stored at 4 °C.

The AS2-US-AuNP-Aggregate kinetic upon interaction with PSA was preliminary investigated by incubating it with PSA at 1 pM and 1 nM concentrations (Figure S4A). Notably, the number-weighted size distribution analysis proved more sensitive than the intensity-weighted size distribution analysis for detecting smaller particles in the sample (Figure S4A; cyan vs. violet dots in Figure S5); these smaller particles likely correspond to single nanoparticles or smaller aggregates released from the AS2-US-AuNP-Aggregates. Smaller particles were observed by DLS at 15–20 min of incubation with PSA at 1 pM concentration, even if they were not always highlighted by the number-averaged size either (Figure S4A and S5C). This occurred more frequently at 1 nM PSA, starting after about 5 min (Figure S4A and S5D). Despite the occasional bigger impact of larger particles after the aggregate started to disassemble, these data indicate a concentration-dependent response. The AS2-Ctrl-US-AuNP-Aggregate, under the same conditions, did not show distinct changes in size over the observed period (Figure S4B), confirming the specificity of the reaction.

To gain deeper insights into the mechanism underlying aggregate disassembly and, in particular, the irregular behaviour observed for the number-weighted size distributions, we conducted a more in-depth analysis of the DLS data. Indeed, the accuracy of the fit provided by the DLS software can be affected by long-time decays in the autocorrelation function, due to sedimentation or movement of large aggregates, dust particles, or bubbles. These can introduce a background signal that affect data interpretation. To mitigate this issue, we performed an additional regression analysis of an autocorrelation function generated by the DLS software, by fitting it with simpler functions from whose parameters we estimated one, two, or three sizes characterising the size distribution of the nanostructures in the observed suspension (Figure S5A; refer to the experimental section for details). The results of this analysis did not contradict the previous results about stability and dose–response behaviour of the AS2-US-AuNP-Aggregates. However, they helped with better visualising the appearance 8–15 min upon PSA introduction of at least two populations of scattering entities characterised by sizes d_1_ and d_2_ (see Figure S5C,D and the experimental section for details). These observations are also supported by analysing the intensity-weighted distributions of sizes at different times after the introduction of different concentrations of PSA in the solution containing the AS2-US-AuNP-Aggregates (Figure S6), which revealed the appearance of a second peak with smaller hydrodynamic radius 8/13 min after PSA addition (Figure S6B,C). Another, simpler way to highlight and quantify this trend was to consider the time average (and standard error) of the DLS number-weighted-average size in a ~5-min range (or more) after 15 min upon PSA addiction (Figure S6D). This approach allows for a faster automatic description of the DLS outputs and is utilised for the presentation of the next results.

**Figure 4:**
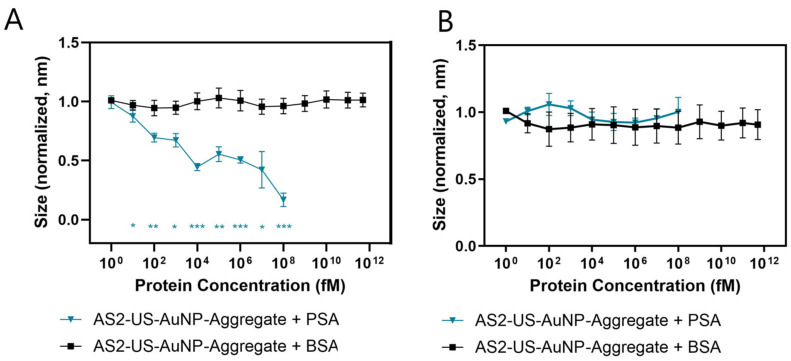
Response to PSA of the AS2-US-AuNP-Aggregates and relative controls. (**A**) Variation in the number-weighted averaged size, measured by DLS, of the AS2-US-AuNP-Aggregate when incubated with different concentrations of the PSA protein (blue line and triangles) or of BSA (black line and squares). (**B**) Size measurements of the AS2-Ctrl-US-AuNP-Aggregate when incubated with different concentrations of the PSA protein (blue line and triangles) or of BSA (black line and squares). All measurements are performed with n = 4, from independent experiments, and the error bars represent the standard errors. * indicates a *p*-value < 0.05, ** indicates a *p*-value < 0.01, *** indicates a *p*-value < 0.001.

The response of the AS2-US-AuNP-Aggregate to PSA was evaluated across a concentration range of 1 fM to 100 nM. Number-weighted means of sizes were averaged over a 5-min interval, beginning 15 min after PSA addition. These average sizes for AS2-US-AuNP-Aggregates decreased upon incubation in a concentration-dependent manner (Figure 4A, blue line and triangles). A statistically significant reduction in the aggregate size was observed starting at a PSA concentration of 10 fM (*t*-test). The AS2-US-AuNP-Aggregate response increases at higher PSA concentrations, as revealed from a further decrease in the detected average size of the nanostructures (Figure 4A and Figure S8A). The specificity of the reaction was tested by incubating the AS2-US-AuNP-Aggregates with BSA up to its physiological concentrations (500 μM). BSA was selected due to its structural similarity to Human Serum Albumin (HSA); both albumins are commonly used in biophysical and biochemical studies [29]. No significant changes were observed in the aggregates size upon BSA incubation, even at the highest tested concentration (Figure 4A, black line). Similarly, the AS2-Ctrl-US-AuNP-Aggregate, under the same conditions, showed no discernible trend either in size variation (Figure 4B) or for plasmonic shift (Figure S7B), neither with PSA nor with BSA.

We checked for changes in the UV-Vis absorbance spectra of AS2-Ctrl- and AS2-US-AuNP-Aggregates upon introduction of PSA in all tested concentrations and conditions. The AS2-Ctrl-US-AuNP-Aggregate never showed a significant plasmon shift at any concentration, as expected. A tendency of the plasmonic peak towards shorter wavelengths was observed upon incubation of the AS2-US-AuNP-Aggregate with increasing concentrations of PSA; however, this trend was not consistently significant. A clearer variation was detectable only at higher PSA concentrations, where the sensitivity of DLS proved more reliable in capturing aggregate disassembly, although even in this case, the trend was not fully consistent (Figure S7A). This may be attributed to the weak plasmonic resonance of US-AuNPs and the limited efficiency of interparticle plasmon coupling within the aggregates [19]. Moreover, larger aggregates are likely to contribute more strongly to UV–Vis measurements; in this context, even a small number of large aggregates may significantly affect the overall spectrum, as their increased scattering and absorbance can mask the contributions of smaller particles.

We are not suggesting to use the methods exploited here in a real clinical sensor to characterise the response of the aggregates to different PSA concentrations; nevertheless, in order to better describe this response, we calculated a sort of calibration curve for both size and plasmon peak position measurements using linear fits in a semilogarithmic scale (Figure S8). In order to calculate a parameter corresponding to a limit of detection, the fluctuations in the measurement results without PSA are not a good estimate for the uncertainty to be used for the LoD calculation, since these measures are used to “normalise” the data reported in Figure 4, Figures S7 and S8 before averaging; for this reason, we extracted an average standard deviation from the fit residuals. In this way, and considering the logarithmic scale for the concentrations, we obtained indicative LoDs of 2.42 pM (from the normalised variation in the number-averaged size) and 1.89 × 10^8^ pM (plasmon shift). Notably, the inconsistent trend of the plasmon shift indicates that it is not reliable for quantitative purposes; for this reason, subsequent experiments focused exclusively on DLS measurements. It is important to emphasise that the LoD values are reported only as reference metrics derived from the fitted data, without implying analytical performance or practical detectability for the conceived sensor based on the studied aggregates.

### 3.5. AS2:RevAS2 dsDNA in Blood-Mimicking Conditions

To accurately evaluate the AS2-US-AuNP-Aggregate stability and response under conditions that mimic in vivo environments, examination at least in plasma, serum, or mimicking conditions is essential. In the bloodstream, the presence and activity of nucleases, along with the formation of a plasma protein corona, likely affects nanoparticles properties [30]. We examined how plasma components affect the stability of the AS2-tail:RevAS2 duplex and the AS2-US-AuNP-Aggregate by comparing the digestion kinetics in the presence of a single nuclease (DNaseI) and in full human plasma. The stability of AS2-tail:RevAS2 sequences in human plasma, or with DNaseI, were estimated using the AS2-tail-Atto580Q:RevAS2-Rhodamine duplex by monitoring the restoration of Rhodamine fluorescence upon nuclease digestion.

Considering the effect of DNAseI alone, we were able to compare the digestion kinetics of the AS2tail:RevAS2 sequences in solution, and when incorporated into the AS2tail-US-AuNP-Aggregates: the stability of AS2-US-AuNP-Aggregates was tested for DNAseI digestion, monitored by size variations via DLS, and compared with the results on AS2-tail-Atto580Q:RevAS2-Rhodamine at the same DNA and DNAseI concentrations (Supplementary Section S2). We obtained a recovery of fluorescence for AS2-tail-Atto580Q:RevAS2-Rhodamine with a half-life of 1.07 ± 0.53 h (Figure S9A), with complete digestion achieved after 2 h. Checking the DLS-measured number-weighted averaged sizes of the AS2-US-AuNP-Aggregates over time, under identical DNA and DNaseI concentrations, we estimated a half-life of 2.1 ± 0.1 h (Figure S9B). This finding indicates that the AS2-US-AuNP-Aggregate provides increased stability to nuclease digestion, as the embedded duplex exhibits almost a two-fold increase in half-life compared to free dsDNA. These results highlight the ability of DNA-driven nanoaggregated architectures to offer a protective effect against deoxyribonucleases activity [31].

Then, we tested the AS2-tail-Atto580Q:RevAS2-Rhodamine duplex in human plasma; full plasma could not be used with the AS2-US-AuNP-Aggregate because the background scattering compromised the reliability of the DLS measurements. Also, in the case of fluorescence measurements, we needed to consider a background arising from the plasma components (see Supplementary Section S3 and Figure S10A). After its subtraction, we obtained an estimated half-life of fluorescence recovery for the AS2-tail-Atto580Q:RevAS2-Rhodamine in 1:8 diluted plasma of 13.2 ± 1.3 h (Figure S10). Adjusting for the dilution factor and considering a first-order kinetics, the estimated half-life in undiluted plasma was approximately 1.65 ± 0.17 h.

Finally, we investigated the stability and PSA response of AS2-US-AuNP-Aggregates in blood-mimicking conditions. However, as stated above, following the AS2-US-AuNP-Aggregate behaviour in whole plasma using DLS is challenging due the presence of similarly sized particles, including small vesicles, lipoproteins, and protein aggregates. These biological components contribute to significant scattering background and interference, complicating the interpretation of the aggregate size distribution measurements. To address this, female human plasma was purified by centrifugation and filtration to remove all these components. Analysis of the untreated human plasma with DLS revealed two main peaks: one at 145.3 ± 5.9 nm and another at 13.2 ± 2.7 nm. The purification left only smaller molecules, including proteins, as confirmed by the presence of a single peak at 15.9 ± 1.3 nm in the centrifuged and filtered plasma (we show a representative intensity-weighted distribution in Figure S11).

**Figure 5:**
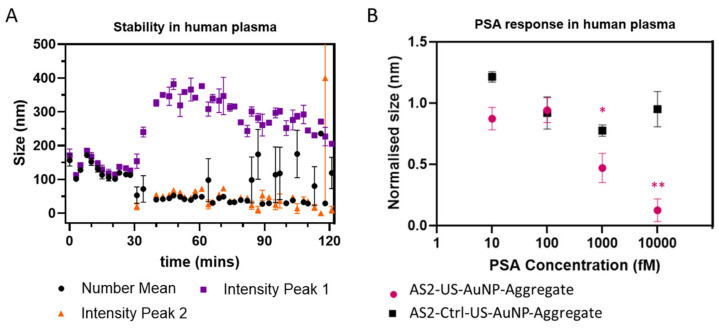
(**A**) Stability profile of AS2-US-AuNP-Aggregate over time in filtered human plasma diluted 1:6. Purple squares represent the average within the most populated peak in the intensity-weighted distribution of the aggregates sizes (Intensity Peak 1), orange triangles indicate the same quantity in the second-most populated peak (Intensity Peak 2, when present), and black dots correspond to size averages weighted in number. Each data point represents the average of three readings acquired within a ~2-min time window, from a single measurement; error bars are standard errors. (**B**) AS2-US-AuNP-Aggregate (pink dots) and AS2-Ctrl-US-AuNP-Aggregate (black squares) size variation before and after 20 min incubation with PSA and filtered plasma. Normalisation is performed with respect to the aggregate number-weighted size before the incubation with PSA. Number-weighted size decreased from 174.6 ± 38.1 nm (prior to PSA incubation) to 92.5 ± 22.6 nm (for 1 pM PSA) and to 49.2 ± 8.7 nm (for 10 pM PSA). Each data point represents the average of three readings acquired within a ~2-min time window, from a single measurement, after 20 min of PSA incubation; error bars are standard errors. T-test was used to compare the aggregate normalised size without PSA with respect to the one after PSA incubation. * indicates a *p*-value < 0.05, and ** indicates a *p*-value < 0.01.

The stability of the AS2-US-AuNP-Aggregates was evaluated in filtered plasma by DLS, as identifying the time window during which the aggregate remains intact is crucial for enabling effective monitoring of the PSA binding reaction. Figure 5A summarises the results, showing the time behaviour of the number-weighted average size and of the intensity-weighted average size on the total distribution. Figure 5A shows that the measured size distributions remain relatively constant during the first ~30 min, with a single peak characterised by a number-weighted size of about 110–160 nm and a slightly higher value for intensity-weighted size distributions (see also Figure S12, black and orange curves), consistent with the stability of the AS2-US-AuNP-Aggregate. After ~30 min, a bipartite distribution of sizes becomes evident in the intensity-weighted size distributions, with two peaks (see also Figure S12, cyan, blue, and violet curves). The smaller peak has an intensity-averaged size around 50 nm or below, with a tendency towards smaller sizes at longer times. This suggests the formation of smaller components, likely due to the disassembly of AS2-US-AuNP-Aggregates upon their interaction with plasma molecules and, in particular, by nuclease digestion. Additionally, the number-weighted averaged size usually follows the position of this peak, confirming that the size derived from the number distribution highlights the presence of smaller particles in the solution. The larger peak in the intensity-weighted distribution initially increases rapidly, reaching a broad maximum at ~350 nm around 50–60 min, suggesting aggregation or fusion with plasma components. Then, it shows a gradual decrease in size, indicative of ongoing erosion of the aggregates (Figure 5A and violet and blue curves in Figure S12). This dynamic behaviour underscores the partial instability of the system beyond 30 min in the plasma environment. Differently from what is observed with DNaseI, the observed half-life of the AS2-US-AuNP-Aggregate in filtered plasma is shorter than the one estimated for the free AS2-tail:RevAS2 duplex in whole plasma. This difference could be caused by differences in the compositions of the two environments (different lots of plasma, treated differently) but anyhow highlights how plasma—a more complex matrix than PBS with DNaseI, even when purified and filtered—affects the aggregate stability differently, likely due to the presence of additional interacting components.

These observations indicate a critical time frame of around 30 min for monitoring PSA before significant structural changes occur. Therefore, we followed the AS2-US-AuNP-Aggregate response to PSA within 20 min upon analyte addition using DLS (Figure S13). PSA was tested across a concentration range from 100 fM to 10 pM, taking into account the clinically relevant nadir points of 150 and 300 fM proposed by Thaxton [5] and Doherty [4], respectively, as well as the classical nadir point of 6 pM. Time-course analysis revealed a concentration-dependent response. At 1 pM PSA (Figure S13C), the lowest concentration at which a statistically significant and consistentreduction in the AS2-US-AuNP-Aggregate size was observed starting at around 8 min, a progressive decrease in size was observed. This size reduction became increasingly distinct over time if compared to the AS2-Ctrl-US-AuNP-Aggregate, with statistically significant differences detected at multiple time points. At 10 pM PSA (Figure S13D), the AS2-US-AuNP-Aggregate exhibited a faster and more pronounced reduction in size, with significant changes detectable as early as at 3 min and becoming more evident over the 20-min monitoring period. These results suggest a concentration-dependent response of the AS2-US-AuNP-Aggregate also in filtered plasma, with higher PSA levels inducing a more rapid and more significant structural change. Figure 5B summarises the results for the number-weighted average size after 20 min of incubation, the longest time point considered to avoid the instability observed in plasma. As already stated, a significant reduction in size of the AS2-US-AuNP-Aggregate was observed starting at 1 pM PSA. These results indicate that the aggregate retains its ability to respond to PSA within a suitable time frame (i.e., before 30 min), even when incubated in a complex environment that mimics physiological blood conditions.

## 4. Discussion

We developed and characterised the AS2-US-AuNP-Aggregate system, which we propose for a future possible ultrasensitive PSA biosensor based on the release of renal clearable nanoreporters from these nanostructures upon recognition of PSA in the bloodstream. This study represents a preliminary step toward this in vivo application, laying the groundwork for evaluating the feasibility and practical constraints of such architectures in real biological contexts.

The developed AS2-US-AuNP-Aggregates demonstrated target-responsive rapid disassembly in buffered solutions, with detectable responses at PSA concentrations as low as 10 fM. Under blood-mimicking conditions, a significant signal change was observed starting at 1 pM PSA, with faster and more pronounced kinetics at higher concentrations (10 pM). These results highlight a detection capability that surpasses many existing commercial point-of-care (PoC) assays, positioning the AS2-US-AuNP-Aggregate as a promising platform for ultrasensitive biomarker sensing in complex media, with potential for real-time monitoring and early disease detection. Our system exhibits a bigger sensitivity threshold than some ultrasensitive diagnostic platforms, such as SiMoA (LoD of ~0.15 fM) [32] or nanoparticle-enhanced ELISAs (LoD ~0.03 fM) [33]. However, our platform with the kinds of measurement used in this work is not intended to compete with these technologies. Instead, the experiments performed here serve as a preliminary proof-of-concept to investigate whether this type of architecture can operate in biologically relevant environments and potentially enable in vivo signal transduction. DLS measurements, intended as a primary investigative tool, provided valuable insights into the aggregate size variation upon interaction with PSA. However, given the limitations of DLS in complex biological matrices—such as signal variability due to polydispersity and interference from plasma components—additional characterisation with higher-resolution or molecular-specific techniques is warranted to confirm and refine these findings.

Given that the AS2-US-AuNP-Aggregate was specifically designed for in vivo applications, optimising its performance and stability in biological environments is essential, and this includes strategies for the temporal control of the aggregate integrity. Previous studies have shown that renal-clearable nanoparticles can be detected in urine one hour after triggerable-nanostructure injection [8,15], with complete renal clearance achieved within 24 h post injection [7,12]. In contrast, our system seems to exhibit instability after ~30 min, highlighting the need for optimisation to extend its lifetime in plasma. This could be achieved through the incorporation of antifouling sequences and the use of modified nucleic acids more resistant to nuclease activity [34]; the last method can be effective in blocking exonuclease activity even upon substitution of a small part of nucleotides, which can be easily achieved, e.g., by using modified primers in the amplification by polymerase chain reaction (PCR) of the oligonucleotides [35,36]. In addition to phosphorothioate backbones and locked nucleic acids [31], simpler modifications can also enhance stability in serum conditions, such as the introduction of PEG coatings [37] or terminal modifications at the 3′ and 5′ ends, e.g., with hexaethylene glycol or hexanediol [31,38].

In a physiological context, nuclease activity, together with dynamic blood flow, could also have positive effects. Blood flow would help to remove the detached particles from the interaction site, effectively favouring the system’s kinetics as if it were an irreversible reaction [39,40]. In this scenario, we speculate two key consequences. First, the reaction would no longer be limited by the concentration of analyte species, but rather by the absolute number of analyte molecules available, potentially enhancing sensitivity. Second, nucleases may promote this irreversibility, by digesting the oligomers on the disassembled nanoparticles faster. On the other hand, as discussed above, the spontaneous instability of the aggregates in plasma—partially influenced by nuclease activity—must be carefully considered. Within the intended reaction time window, the system must remain stable long enough for PSA recognition, subsequent aggregates disassembly, and excretion of the disassembled nanoparticles to urine for detection. However, over longer timescales, complete nuclease digestion could be beneficial, ensuring full disassembling and clearance of nanoparticles also in the absence of PSA, an essential requirement for safe in vivo applications.

The use of US-NPs-Aggregate systems could enable in the future the translation of a specific protein biomarker’s presence into a measurable reporter signal in urine; indeed, the size of the US-AuNPs was specifically optimised to enable renal clearance, thereby allowing downstream urinary analysis. We propose that techniques such as inductively coupled plasma mass spectrometry (ICP-MS) offer a promising and highly sensitive approach for detecting and quantifying these nanoparticles in urine samples. However, other kinds of reporters (e.g., different nanoparticles) and/or detection techniques can be considered using the same kind of DNA architecture proposed here; moreover, the design of the proposed nanostructure could be adapted for the detection of other disease-associated biomarkers by considering different aptamers. These generalisations can broaden the potential applications of the proposed architecture in diagnostic medicine.

## 5. Conclusions

This work presents a preliminary investigation on US-NP-Aggregate systems for transducing the presence of a protein biomarker into measurable urinary signals. We developed the AS2-US-AuNP-Aggregate for ultrasensitive PSA detection after radical prostatectomy. When tested in buffer conditions, the AS2-US-AuNP-Aggregate exhibited a rapid PSA-induced disassembly and achieved ultrasensitive detection levels, with a significant response at 10 fM in PBS, compatible with the USPSA. When transitioning to blood-mimicking conditions, the system behaviours changed. The aggregate stability is compromised by plasma components (nucleases and protein) and the reaction kinetics slow down. We selected a measurement time of 20 min as a compromise between signal development and aggregate stability, achieving a detectable signal at 1pM, still superior to existing PoC approaches.

DLS outperformed plasmonic spectroscopy for this system, since the US-AuNP exhibits a weak plasmonic resonance, and the plasmonic shift is small when assembled in the aggregates (and even smaller upon partial disaggregation), probably because of weak electromagnetic coupling in the aggregates. Moreover, components in plasma produce a high background that hinders plasmon peak position measurement. In contrast, although DLS was used only as a preliminary tool in this investigation, it provided a clearer and more reliable readout of the PSA-triggered disassembly process, even in complex matrices. The limited stability in plasma suggests the need for additional optimisation before in vivo implementation. Nucleases and protein binding influence both the kinetics and the structural integrity of the system, potentially affecting its sensitivity and integrity. Strategies such as the incorporation of antifouling sequences or nuclease-resistant nucleic acid could enhance the aggregate stability and performance.

## Data Availability

Data presented in this study is contained within the article. Further inquiries can be directed to the corresponding authors.

## Supplementary Materials

The following supporting information can be downloaded at: https://www.mdpi.com/article/10.3390/s26010033/s1, Results S1: AS2 ELONA analysis; Results S2: AS2-tail-Atto580Q:RevAS2-Rhodamine dsDNA digestion in plasma analysis; Results S3: AS2-tail-Atto580Q:RevAS2-Rhodamine dsDNA digestion by DNaseI analysis; Figure S1: aPSA and AS2 ELONA; Figure S2: Secondary folding of the AS2-ctrl strand and of the AS2-ctrl:RevAS2 duplex; Figure S3: Absorbance spectra and plasmon peak analysis of non-functionalised US-AuNPs; Figure S4: Stability and kinetic analysis of the aggregates in buffer; Figure S5: Comparison between DLS extracted data and the analysed data with the multiexponential fit; Figure S6: Intensity-weighted DLS distributions for the AS2-US-AuNP-Aggregate with and without PSA at different incubation times; Figure S7: Plasmon shift variation in the AS2-US-AuNP-Aggregate in response to PSA presence and relative control; Figure S8: Calibration curves of the AS2-US-AuNP-Aggregate in response to PSA; Figure S9: AS2:RevAS2 digestion analysis in plasma; Figure S10: Comparison of digestion kinetics with DNaseI of AS2:RevAS2 and AS2-US-AuNP-Aggregate; Figure S11: Representative DLS measurements of plasma before, during, and after processing; Figure S12: Representative DLS measurements for AS2-US-AuNP-Aggregate stability in plasma; Figure S13: AS2-US-AuNP-Aggregate and AS2-Ctrl-US-AuNP-Aggregate number-weighted size variation in time during PSA incubation. Table S1: DNA sequences used in this study; Table S2: Melting temperatures of the PSA and AS2 group of sequences.

## Abbreviations

The following abbreviations are used in this manuscript:

BCR	Biochemical Recurrence
BSA	Bovine Serum Albumin
DLS	Dynamic Light Scattering
ELONA	Enzyme-Linked Oligonucleotide Assay
EPR	Enhanced Permeability and Retention
HAuCl_4_	Chloroauric Acid
HRP-streptavidin	Horseradish Peroxidase–Conjugated Streptavidin
HPLC	High-Performance Liquid Chromatography
HSA	Human Serum Albumin
LFA	Lateral Flow Assay
LoD	Limit of Detection
