# Supplementary material for: PSA-Responsive Aptamer-Based Switchable Aggregates of Ultrasmall Gold Nanoparticles
Source: Sensors (Basel). 2025 Dec 20;26(1):33. doi: 10.3390/s26010033 (PMC12787490; doi:10.3390/s26010033)
Supplement: Supplementary file 1 [file sensors-26-00033-s001.zip › sensors-3994742-supplementary.pdf]

Supplementary Results

S1. AS2 ELONA

As stated in the main text and illustrated in Fig. S1C, the first concentration at which the ELONA assay of the AS2-biotin aptamer showed a significant difference with respect to the background was 250 nM, way higher than the dissociation constant reported in the original work where the sequence was developed (700 fM) [1] .This difference may be attributed to variations in buffer composition relative to the conditions employed during aptamer selection and development (2 mM $MgCl_2$ and 0.02% Tween-20 in PBS, pH 7.4, versus PBS supplemented with 5 mM $MgCl_2$ in our protocol), as well as to the inherent limitations of the direct ELONA assays, which tend to be less sensitive than in the sandwich configuration. In the direct configuration, proteins adsorb in a random manner on the well surface, potentially concealing a significant portion of the epitopes recognised by the aptamer. This could lead to a considerable reduction in the sensitivity also by several orders of magnitude [2]. Moreover, we don't have any information about the equivalent concentration of the adsorbed proteins, and this data could influence the measured response in ELISA and ELONA. Despite this consideration, with the reported ELONA tests we could determine at least qualitatively that the AS2 aptamer is able to bind the PSA protein in physiological conditions (more than the aPSA), and that the hybridization with the RevAS2 sequence did not change substantially this property (Figure S1A).

S2. AS2:RevAS2 dsDNA digestion by DNase I

The stability of AS2-US-AuNPs-Aggregates was evaluated against nucleases digestion by monitoring size variations via DLS upon the addition of DNase I. An AS2-US-AuNPs-Aggregate solution with a final DNA concentration of 20 nM—the same concentration used in fluorescence recovery assays in plasma—did not produce a measurable DLS signal. To enable a direct comparison between nuclease digestion of DNA duplexes in plasma and

DNase I digestion in buffer, the concentrations of both DNA duplexes and AS2-US-AuNPs-Aggregates were increased by a factor of 10 for these measurements. Thus, the digestion experiments were conducted using 200 nM AS2-tail-Atto580Q:RevAS2-Rhodamine duplex and the corresponding AS2-US-AuNPs-Aggregates at an equivalent DNA concentration.

The digestion of AS2-US-AuNPs-Aggregates was tracked by observing changes in both the number-weighted-averaged hydrodynamic size (Figure S9B black circles) and the DLS count rate (Figure S9B white squares). Size measurements indicated rapid digestion within the first 4 hours, with digestion reaching completion after approximately 10 hours. In contrast, the count rate showed a different pattern: it decreased slightly during the first 2 hours post-DNase I addition, then remained relatively stable or even exhibited a slight increase after 5 hours. This discrepancy likely reflects the dual dependence of the scattering intensity on nanoparticle size and particle number within the colloidal suspension, as digestion reduces aggregate size but increases the number of smaller scattering particles. Since the electromagnetic interactions between the US-NPs inside aggregates are low, they scatter light with an intensity similar to isolated US-NPs (and not with a larger intensity as expected in the case of a strong electromagnetic coupling), so no or little change in scattering intensity is expected upon disaggregation.

S3. AS2-tail:RevAS2 dsDNA digestion in plasma - Analysis

The digestion kinetics of AS2-tail:RevAS2 annealed sequences was estimated using the AS2-tail-Atto580Q:RevAS2-RhodamineB duplex by monitoring the restoration of Rhodamine fluorescence upon nuclease digestion. The fluorescence recovery was observed in 1:8 diluted human plasma in 10-30 hours. However, a high background signal was detected, likely due to stray-light from the excitation source in a highly light-scattering sample, as well as to plasma autofluorescence.

The background trend was determined for each series of measurement by averaging all the measured fluorescence spectra and fitting with a double exponential the data between 554 and 560 nm and between 670 and 710 nm (Figure S10A). For each spectrum, an addictive

and a multiplicative constant for this background have then be determined for its subtraction; these parameters were determined by minimizing the squared sum of the residuals in the same intervals (Figure S10B). In some of the shown result, the obtained Rhodamine B fluorescence spectrum (and therefore its integral) was corrected by dividing by the multiplicative constant, on the basis that changes in the intensity of emission or stray-light diffusion by the plasma components could be caused mostly by water evaporation, and they would apply in the same way also to the measured Rhodamine B fluorescence.

Beyond 20 hours, measurements became less reproducible and data for these time points were not recorded for all samples. However, all series appeared to reach a plateau towards the end of the measurement period (Figure S10C). To quantify the half-life of AS2-tail-Atto580Q:RevAS2-Rhodamine DNA digestion in diluted plasma, we plotted all the acquired data, averaged at time points when more than a measurement was done, and we carried out an unweighted fit with an exponential function (Figure S10D). The estimated half-life in 1:8 diluted plasma was 13.2±1.3 hours. Adjusting for the dilution factor, the estimated half-life in undiluted plasma would be approximately 1.65±0.17 hours.

Supplementary Figures and Tables

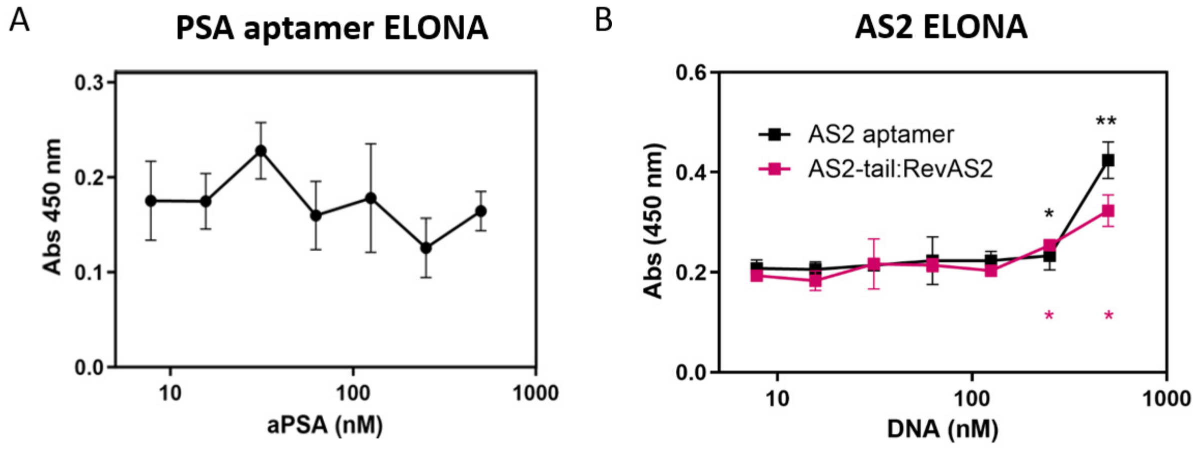

Figure S1 (A) ELONA assay with the aPSA-biotin aptamer did not show any significant response curve. **(B)** ELONA assay of the AS2-biotin aptamer (black line and squares) and the AS2-tail-biotin:RevAS2 (red line and squares) show a concentration-dependent response. Red and black stars indicate the aptamer concentrations causing a signal significantly different from the blank case (no aptamers) for A2:RevAS2 and for the AS2 aptamer, respectively. * indicates a p value < 0.05 and ** indicates a p-value < 0.01. Statistics is t-test with respect to blank. All measurements were repeated in triplicate and the error bars are standard errors.

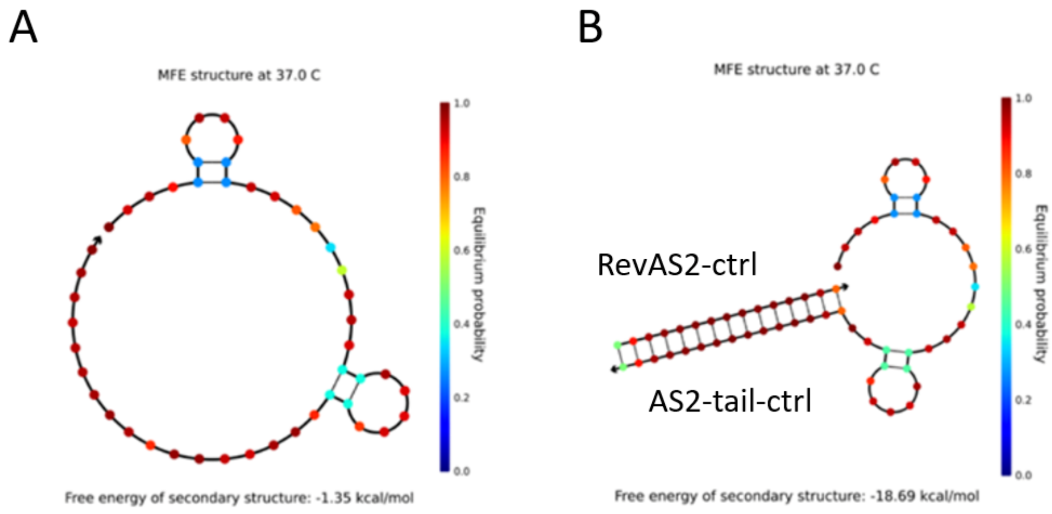

Figure S2 Secondary Folding of: **(A)** the AS2-tail-ctrl strand; **(B)** the AS2-tail-ctrl:RevAS2-ctrl duplex. The colours code for the probability of base pairing at equilibrium (i.e. the probability of the corresponding base pair forming) according to the colour scale bar reported at the right of each panel. The simulation was performed with NUPACK online tool at the following conditions: DNA concentration 100 nM, Temperature 37°C, NaCl concentration 0.15 M and Mg^{2+} concentration 0.025 M.

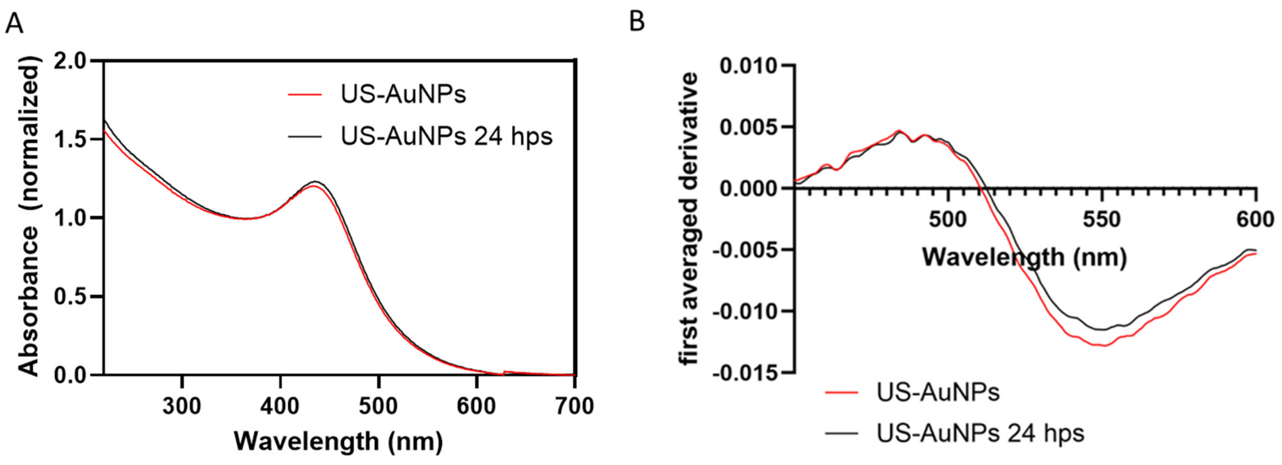

Figure S3: (A) Normalized absorbance spectra of US-AuNPs at 450 nm, for non-functionalized US-AuNPs immediately after synthesis (red line) and 24 hours post-synthesis (24 hps, black line). **(B)** The plasmon peak position was determined by the zero of the first averaged derivative of the spectra shown in panel **(A),** reported here with corresponding colours. The plasmon peak wavelength shifts from 510 to 512 nm, suggesting an aggregation of US-AuNPs.

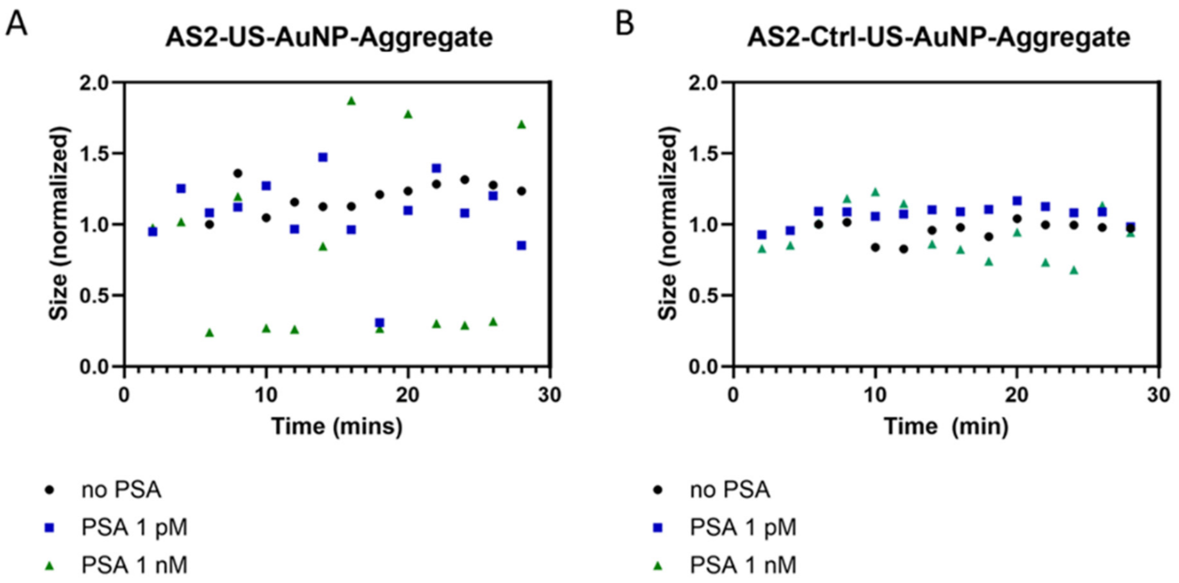

Figure S4: Stability in pure buffer (black dots) and kinetic analysis of the Aggregates incubated with 1pM (blue squares) or 1nM of PSA (green triangles). **(A)** Normalised number-averaged size variation of the AS2-AuNPs-Aggregate recorded at DLS; **(B)** Normalised number-averaged size variation for the AS2-Ctrl-US-AuNPs-Aggregates. The sizes when Aggregates are incubated with PSA are normalized with respect to their size measured before PSA addition. Data obtained from single samples.

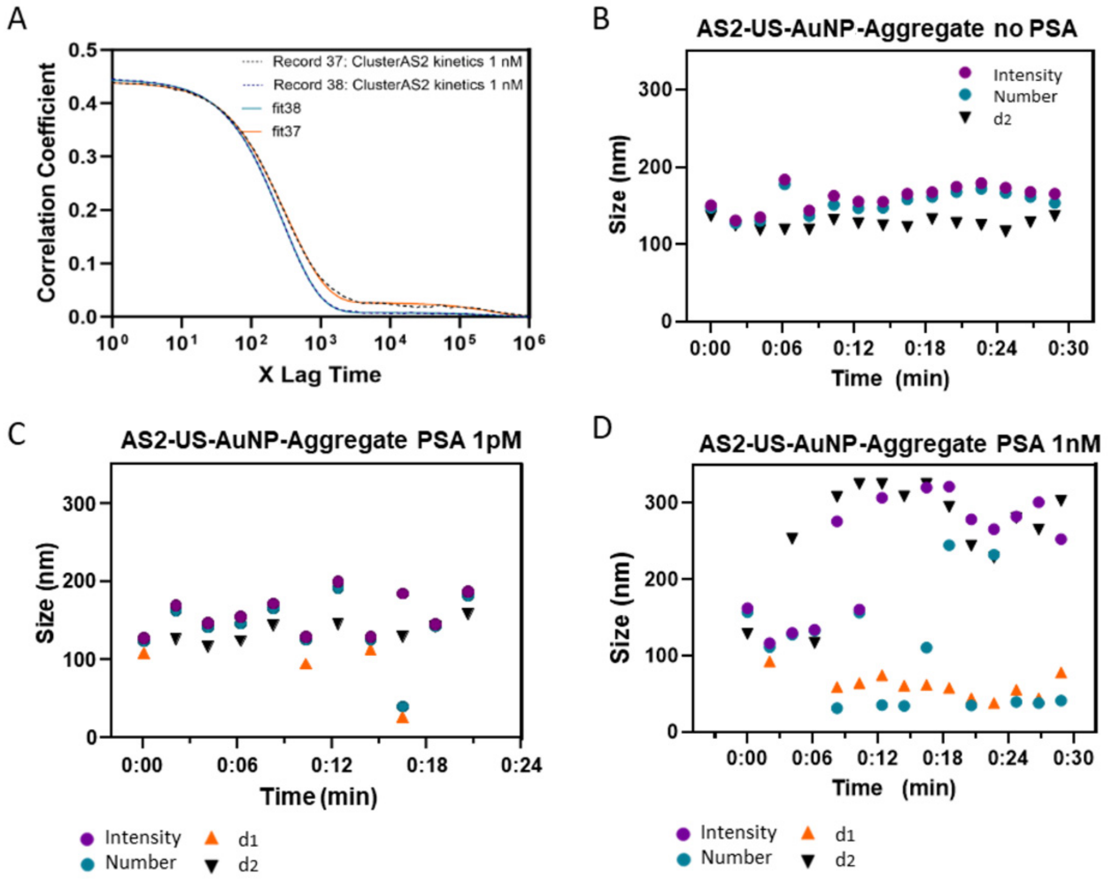

Figure S5 (A) Examples of correlation coefficient correlograms (proportional to $|g^1(\tau)|^2$, continuous lines; output from DLS software) and results of our squared multiexponential fit (short-dashed lines): in this case, the long-time noisy background is fitted better than in the DLS software, assuring more trustworthy parameters also for the shorter fitted decay times. The data arise from samples incubated with 1 nM of PSA at two different sequential times. **(B, C, D)** Comparison between DLS extracted data, which automatically provides "Intensity" (intensity-weighted average diameters, purple dots; the result for the most populated peak is reported if more than one were detected) and "Number" (number-weighted average diameters, cyan dots) sizes, and the results of the analysis exemplified in panel A; d_1 corresponds to characteristic sizes between 0 and 116 nm and d_2 to characteristic sizes between 116 and 325 nm, and are shown only if their weight c_i (see Experimental section in the main text) is bigger than a threshold (2% of the sum of all the weights c_i for a fit with 3 components). Panel B shows the results for the AS2-US-AuNPs-Aggregate without PSA, demonstrating its stability at a temperature of 37 °C over a time period of 30 minutes. Panels C and D represent respectively the Aggregate with PSA 1pM and with PSA 1 nM. Data in panel B, C, and D are obtained from a single sample each.

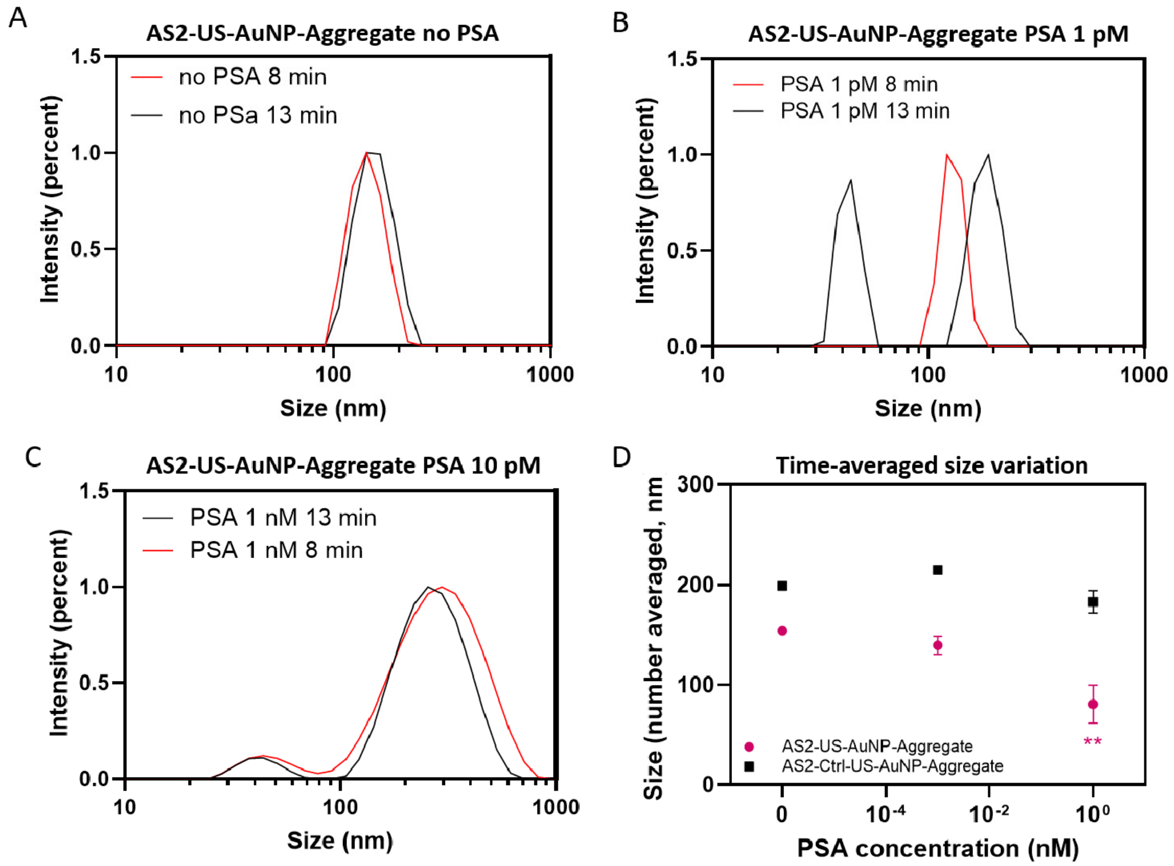

Figure S6 Representative intensity-weighted distributions for the measured diameter of the AS2-US-AuNPs-Aggregate without PSA **(A)**, with PSA at 1 pM **(B)**, and with PSA at 1 nM **(C)**, respectively, at 8 minutes (red line) and 13 minutes (black line after introduction of PSA). **(D)** Size variation of the AS2-US-AuNPs-Aggregate (pink dot) and of AS2-Ctrl-US-AuNPs-Aggregate (black squares) when not incubated with PSA or with PSA at concentrations 1pM and 1nM. The size values represent the average for a single sample measured over a time window from 15 to approximately 20 minutes following PSA incubation, with error bars indicating the standard error of the mean. T-test was used for comparing the Aggregate size without PSA with respect to the one measured after PSA incubation. * indicates a p-value < 0.05 and ** indicates a p-value <0.01

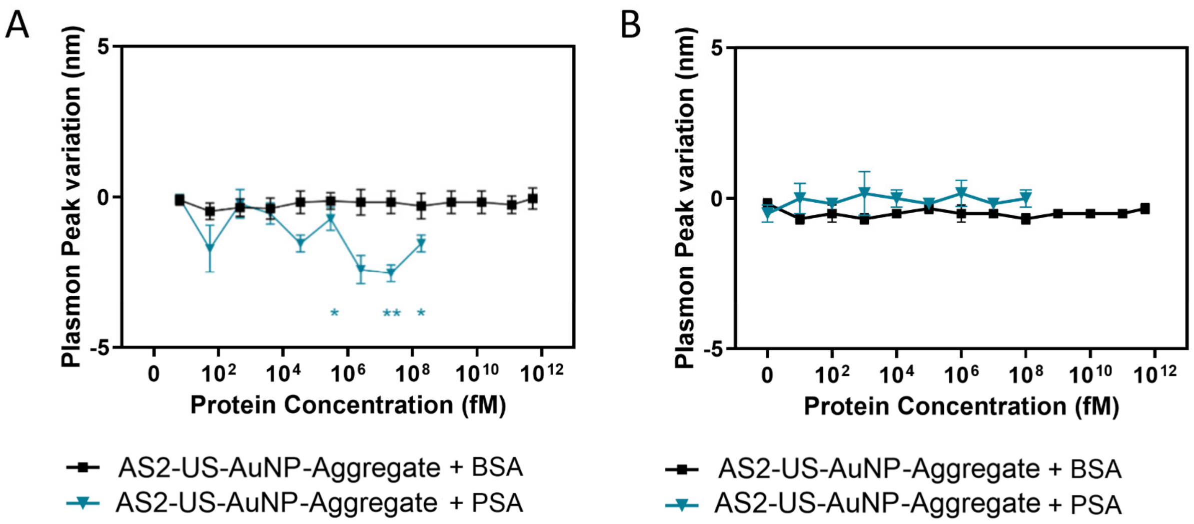

Figure S7 Plasmon shift variation of the AS2-US-AuNPs-Aggregates in response to different PSA concentrations, and relative controls. **(A)** Variation of the plasmonic peak position of the AS2-US-AuNPs-Aggregate when incubated with PSA (blue triangles and line) or with BSA (black line). **(B)** Plasmon peak shift of the AS2-Ctrl-US-AuNPs-Aggregate when incubated with PSA (blue triangles and line) or with BSA (black squares and line). All measurements were performed in n=4 independent experiments, and the error bars represent the standard errors. * indicates a p-value < 0.05 and ** indicates a p-value <0.01, t-test.

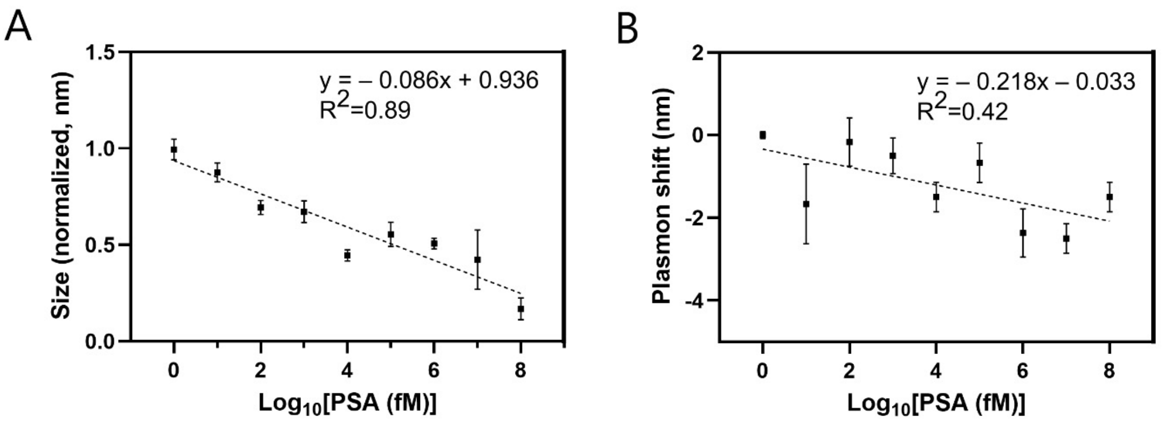

Figure S8 Calibration curves of the number-averaged size-normalised variation (A) and of the plasmon shift (B) of the AS2-US-AuNPs-Aggregate upon incubation with PSA. Both curves are fitted with a linear function on a semilogarithmic scale, x=Log_{10}([PSA], fM). Error bars represents standard errors, n=4.

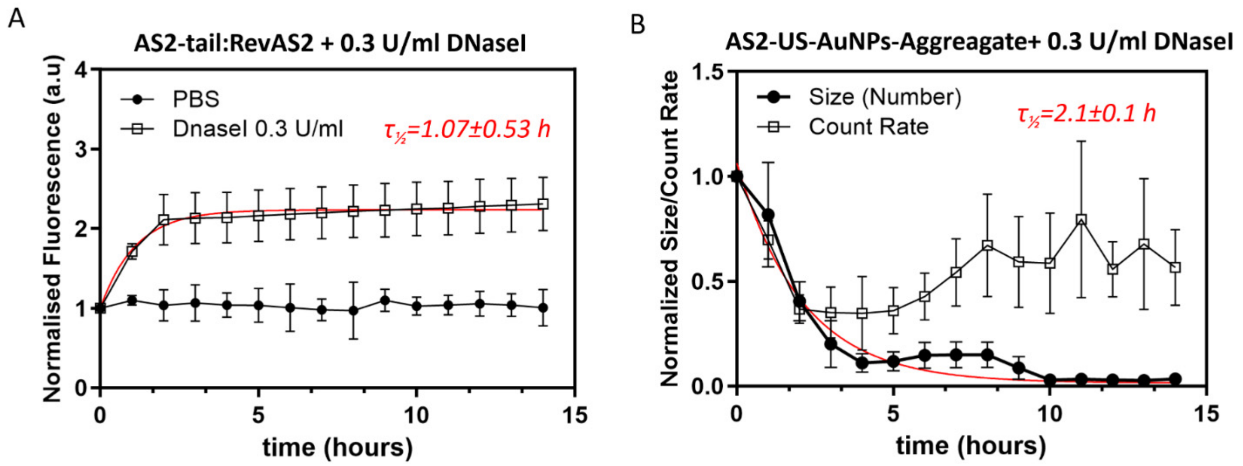

Figure S9. Comparison of digestion kinetics with DNase I. **(A)** Fluorescence recovery of the quenched Rhodamine for AS2-tail-atto580Q:RevAS2-Rhodamine DNA duplex solution digested with DNase I (white squares) and in PBS buffer (black dots). Fluorescence recovery was measured as AUC between λ=570 nm and λ=610. Red line is an exponential fit of the data in the presence of DNase I **(B)** Variation of normalised number-weighted-average size (black dots) and Count Rate (white squares) of the AS2:RevAS2 AuNPs-US-Aggregates when incubated with DNase, as monitored with DLS. The data are normalized to the value immediately before DNase addition (for aggregate sizes, 246.4 ± 29.8 nm). The final aggregate size after digestion was 17.7 ± 1.6 nm. All measurements were performed in triplicates (n=3) and error bars are standard errors. Red line is an exponential fit of the size data.

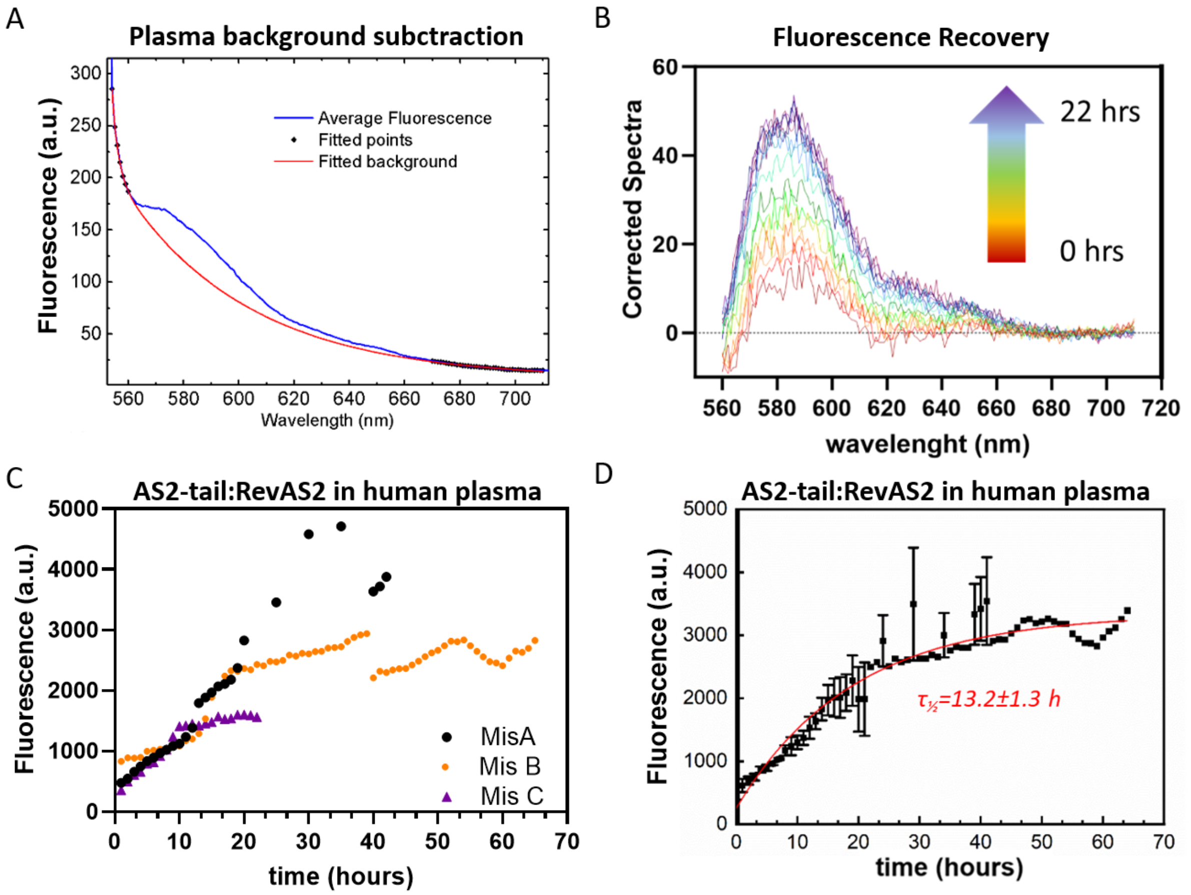

Figure S10. (A) Blue line: average of the spectra from the AS2-tail-Atto580Q:RevAS2-Rhodamine DNA duplex (20 nM) incubated with diluted plasma in a 19-h time series; black diamonds: points of the averaged spectrum used to fit the background; red line: biexponential-with-offset fit on the black diamonds. **(B)** Spectra after background subtraction; time is increasing from 0 to 22 hours for curves from red to violet. The graph illustrates the increase in fluorescence signal over time, indicating DNA digestion from plasma nucleases and subsequent decrease of Rhodamine B quenching. **(C)** AS2-tail-atto580Q:RevAS2-Rhodamine DNA duplex fluorescence recovery when incubated with plasma diluted 1:8. Fluorescence recovery is reported as the integral of the fluorescence intensity between λ=570 nm and λ=610 nm after background subtraction. Here we report the results from three single measurements, each one from an independent preparation. **(D)** Averages from the results reported in panel C after correction for the background multiplicative constant. Measurements after 21 hours showed high variability, and averages are made on $1 \leq n \leq 3$ points. Red dashed line is an exponential fit with half-life $\tau_{1/2}$=13.2±1.3 s (uncertainty from the fit result). Error bars are standard errors, not reported when n=1.

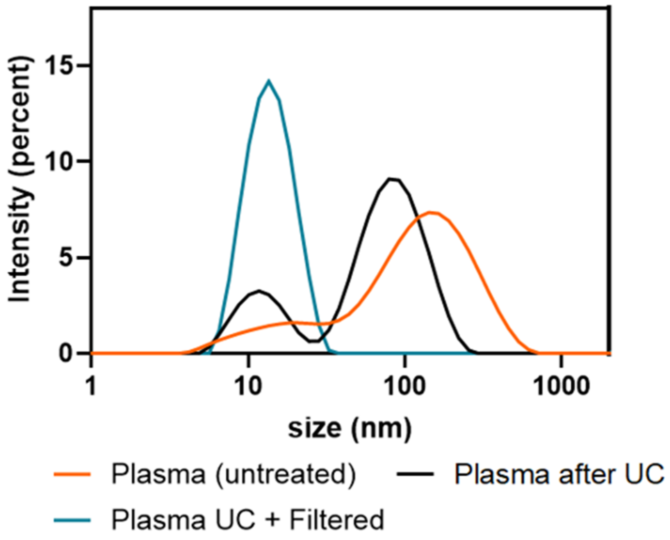

Figure S11. Representative DLS measurements of female human plasma before, during, and after processing. The orange curve refers to the untreated plasma and shows two main peaks in the intensity-weighted distribution, at 161.4 nm and 15.7 nm. The black curve shows the distribution after the last ultracentrifugation (UC), with the larger peak shifted to 90.6 nm and the smaller peak at 12.5 nm, indicating the removal of larger vesicles. The blue curve results from plasma after both ultracentrifugation and filtration, showing a single peak at 14.4 nm.

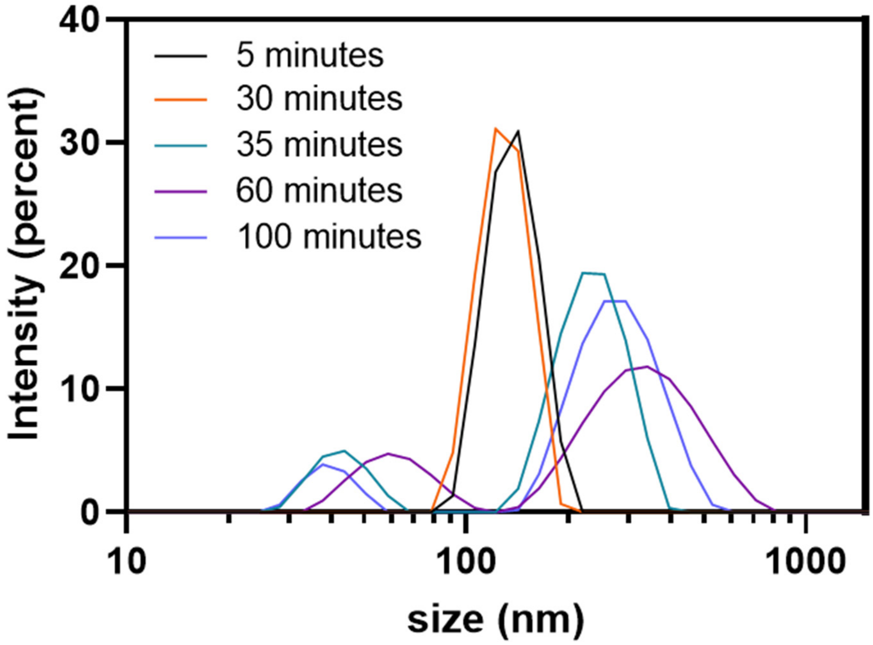

Figure S12. Representative intensity-weighted distributions for the sizes measured for AS2-US-AuNPs-Aggregates at 5 minutes (black line), 30 minutes (orange line), 35 minutes (light blue line), 60 minutes (light purple line) and 100 minutes (dark purple line) after their insertion in 1:6 diluted UC +filtered human plasma.

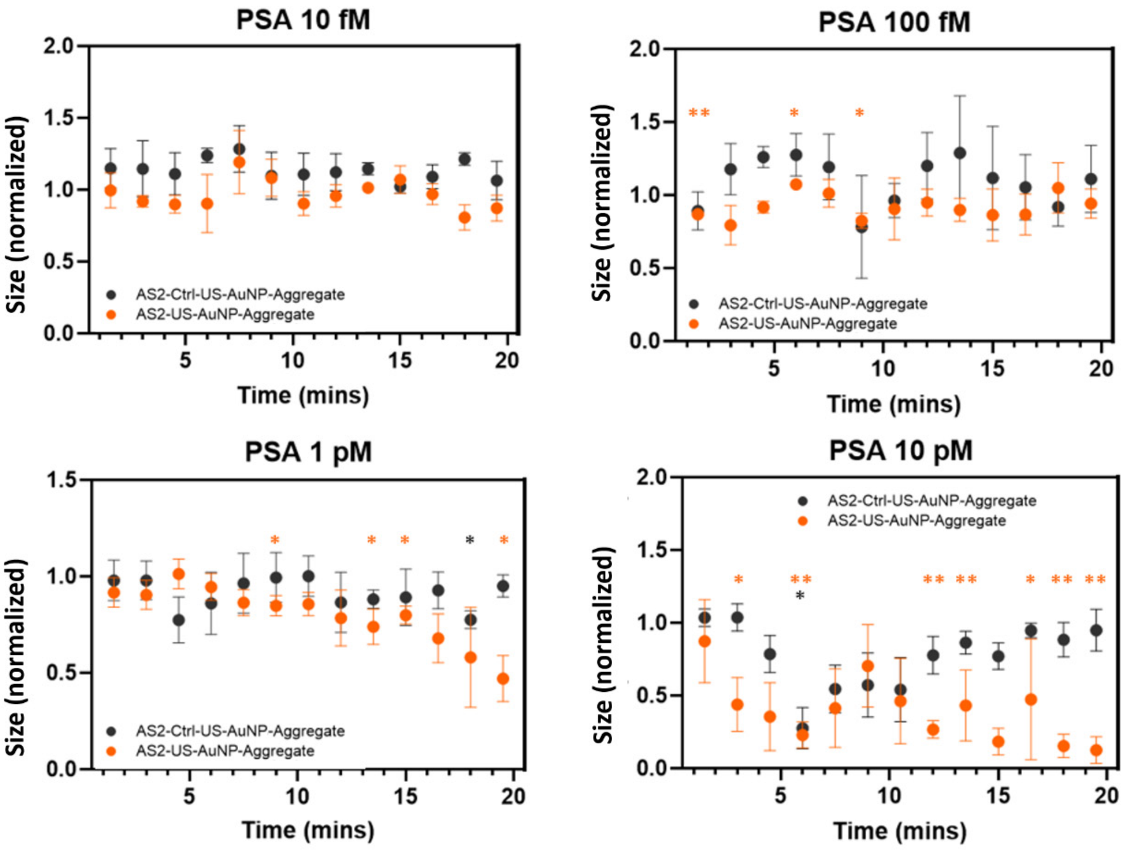

Figure S13. AS2-US-AuNPs-Aggregate (orange dots) and AS2-Ctrl-US-AuNPs-Aggregate (black dots) number-weighted-averaged size variation in 20 minutes of incubation with PSA and plasma proteins. PSA was tested at different concentrations: 10 fM **(A),** 100 fM **(B),** 1 pM **(C)** and 10 pM **(D)**. * indicates a p - value of <0.05 and ** indicates a p-value < 0.01, as from t-tests with respect to 1. All measurements were repeated in triplicate (n=3) from the same AS2-US-AuNPs-Aggregate batch, error bars are standard errors.

Table S1: DNA sequences used in this study. For the AS2 aptamer and its derived sequences, the aptamer sequence is indicated in **bold**, the additional tail in AS2tail is in normal text, and the complementary strands between RevAS2 and AS2tail (and in the control Ctrl) are underlined. Thiol modifier is actually a C6 SS or C3 SS.

	**Mod 5′**	**Sequence**	**Mod 3′**
aPSA		ATT AAA GCT CGC CAT CAA ATA GC	
AS2		**GGG CGG GGC GGA CGA GAC AGT AAG GGC TGT GGG TGT GGT G**	
AS2-biot		**GGG CGG GGC GGA CGA GAC AGT AAG GGC TGT GGG TGT GGT G**	Biotin
AS2-tail-biot		**GGG CGG GGC GGA CGA GAC AGT AAG GGC TGT GGG TGT GGT G**TT TTT TT	Biotin
AS2-tail		**GGG CGG GGC GGA CGA GAC AGT AAG GGC TGT GGG TGT GGT G**TT TTT TT	
AS2-tail-NH$_2$		**GGG CGG GGC GGA CGA GAC AGT AAG GGC TGT GGG TGT GGT G**TT TTT TT	Amine
AS2-tail-thio		**GGG CGG GGC GGA CGA GAC AGT AAG GGC TGT GGG TGT GGT G**TT TTT TT	Thiol
RevAS2		AAA AAA ACA CCA CAC	
RevAS2-NH$_2$	Amino	AAA AAA ACA CCA CAC	
RevAS2-thio	Thiol	AAA AAA ACA CCA CAC	
AS2-tail-ctrl		GAG GCG TGG TCG GAG GCG GAT CGG TAG GCG TG G AGG TAG GTT TTT TT	
RevAS2-ctrl		AAA AAA ACC TAC CTC	
RevAS2-short		AAA AAA ACA	

hlyQF		CAT GGC ACC ACC AGC ATC T	
hlyQR		ATC CGC GTG TTT CTT TTC GA	
L23SQF		AGG ATA GGG AAT CGC ACG AA	
L23SQR		TTC GCG AGA AGC GGA TTT	

Table S2: Melting temperatures (T_ms) of the AS2 group of sequences, as determined in silico from simulations in NUPACK, in vitro from the absorbance reading at 260 nm of the dsDNA sequences, and for Aggregates from DLS experiments (Aggregate T_m). The uncertainty is estimated to be ±0.3 °C, given that the precision of the measurements is ±0.5 °C and they are in triplicates.

**Sequence**	***In silico* T_m (°C)**	***In vitro* T_m (°C)**	**DLS Aggregate T_m (°C)**
**AS2-tail:RevAS2**	56.0	52.0	62.5
**AS2-tail-ctrl:RevAS2crtl**	53.0	53.0	63.8

Table S3: Diameters, plasmon peaks and zeta potentials of naked US-AuNPs, ssDNA functionalised US-AuNPs and US-AuNPs-Aggregates. Data reported are means ± standard errors, but with minimum uncertainty of 0.1 nm and 0.1 mV for Mean Size and Zeta Potential. Minimum Plasmon Peak uncertainty is considered to be ±0.3 nm, given that the precision of the measurements is 0.5 nm and the measurements are in triplicates.

	**Mean Size (Number) (nm)**	**Plasmon Peak (nm)**	**Zeta Potential (mV)**
US-AuNPs	4.6±0.2	508.0±1.0	-11.5±1.3
AS2-tail-US-AuNPs	11.9±0.4	517.5±0.3	-19.7±0.5
RevAS2-US-AuNPs	7.7±0.4	515.3±0.3	-18.8±1.8
AS2-Ctrl-US-AuNPs	15.6±1.3	518.5±0.5	-18.6±0.4
RevAS2ctrl-US-AuNPs	10.0±1.2	517.5±0.5	-28.1±0.1
AS2-US-AuNPs-Aggregate	189±35.3	526.5±0.5	-22.8±0.9
AS2-Ctrl-US-AuNPs-Aggregate	157.5±31.6	528.3±0.5	-20.4±1.7

Supplementary References

1. Park, J.W.; Lee, S.J.; Ren, S.; Lee, S.; Kim, S.; Laurell, T. Acousto-Microfluidics for Screening of SsDNA Aptamer. *Sci. Rep.* **2016**, *6*, 1–9, doi:10.1038/srep27121.

2. Kimoto, M.; Shermane Lim, Y.W.; Hirao, I. Molecular Affinity Rulers: Systematic Evaluation of DNA Aptamers for Their Applicabilities in ELISA. *Nucleic Acids Res.* **2019**, *47*, 8362–8374, doi:10.1093/nar/gkz688.
